# Two new deep-reef basslets (Teleostei, Grammatidae, *Lipogramma*), with comments on the eco-evolutionary relationships of the genus

**DOI:** 10.3897/zookeys.638.10455

**Published:** 2016-12-07

**Authors:** Carole C. Baldwin, D. Ross Robertson, Ai Nonaka, Luke Tornabene

**Affiliations:** 1 Department of Vertebrate Zoology, National Museum of Natural History, Smithsonian Institution, Washington, DC 20560; 2 Smithsonian Tropical Research Institute, Balboa, Republic of Panama

**Keywords:** Manned submersible, cryptic species, integrative taxonomy, phylogeny, ocean exploration, Smithsonian Deep Reef Observation Project (DROP)

## Abstract

The banded basslet, *Lipogramma evides* Robins & Colin, 1979, is shown to comprise two species: *Lipogramma evides*, which inhabits depths of 133–302 m, and a new species described here as *Lipogramma levinsoni*, which inhabits depths of 108–154 m and previously was considered to represent the juvenile of *Lipogramma evides*. A second new species of banded basslet, described here as *Lipogramma haberi*, inhabits depths of 152–233 m and was previously not reported in the literature. Morphologically, the three species differ in color patterns and modal numbers of gill rakers, whereas various other morphological features distinguish *Lipogramma levinsoni* from *Lipogramma evides* and *Lipogramma haberi*. DNA barcode data and multilocus, coalescent-based, species-delimitation analysis support the recognition of the three species. Phylogenetic analysis of mitochondrial and nuclear genetic data supports a sister-group relationship between the two deepest-living of the three species, *Lipogramma evides* and *Lipogramma haberi*, and suggests that the shallower *Lipogramma levinsoni* is more closely related to *Lipogramma anabantoides* Böhlke, 1960, which inhabits depths < 120 m. Evolutionary relationships within *Lipogramma* thus appear to be correlated with species depth ranges, an eco-evolutionary pattern that has been observed in other Caribbean marine teleosts and that warrants further investigation. The new species represent the eleventh and twelfth new fish species described in recent years from exploratory submersible diving in the Caribbean in the globally poorly studied depth zone of 50–300 m. This study suggests that there are at least two additional cryptic species of *Lipogramma*, which are being analyzed in ongoing investigations of Caribbean deep-reef ecosystems.

## Introduction

**Figure 1.**
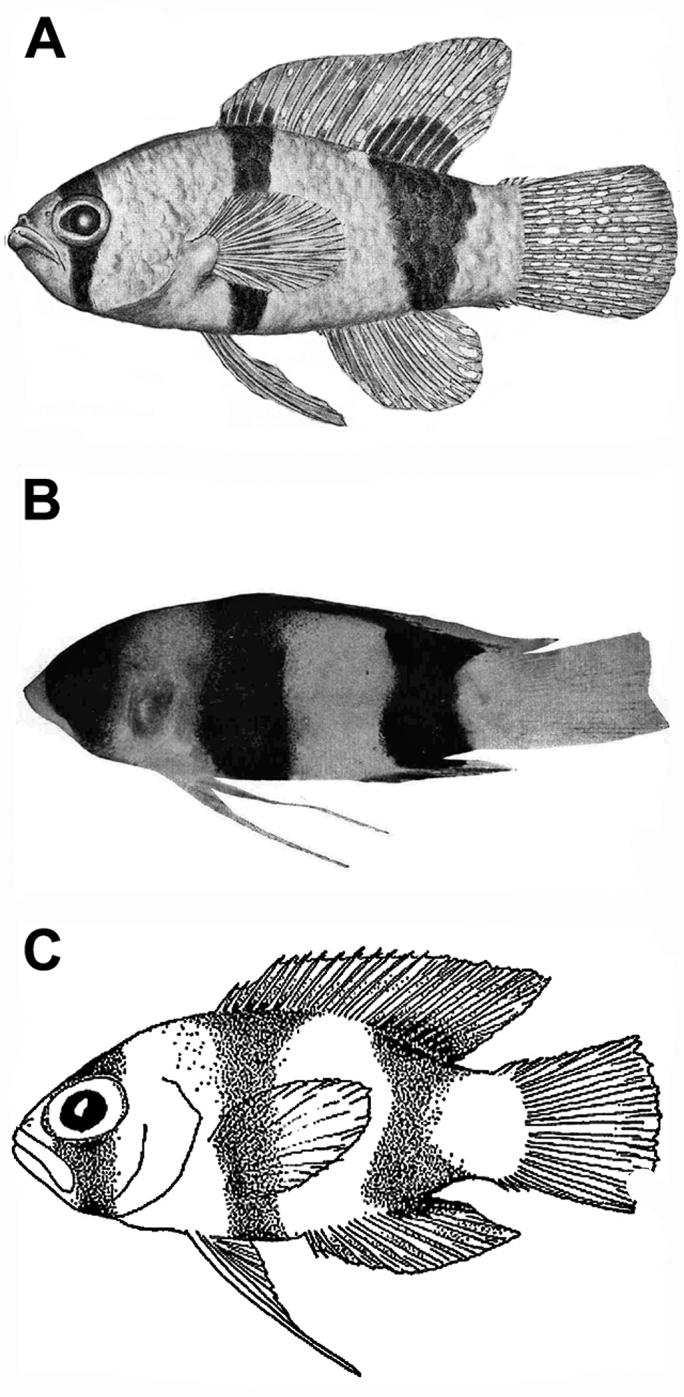
Previously published images of **A** *Lipogramma evides*, 34.4 mm SL, ANSP 134329, holotype, from Robins and Colin (1979: fig. 1) **B** *Lipogramma levinsoni* sp. n., 12.6 mm SL, ANSP 134332, as juvenile paratype of *Lipogramma evides* in Robins and Colin (1979: fig. 2) **C** *Lipogramma levinsoni* sp. n., 14.1 mm SL, IRCZM 107: 07660, as juvenile of *Lipogramma evides* in Gilmore and Jones (1988: fig. 3). Images reproduced with permission from Bulletin of Marine Science.

The western Atlantic family Grammatidae comprises small, usually brightly colored fishes in two genera, *Gramma* with four species and *Lipogramma* with eight (Robertson and Van Tassell 2015). Among other characters, the two genera are distinguished by the absence of a lateral line and presence of thickened, spinous, outer procurrent rays in *Lipogramma* (Mooi & Gill, 2002). The Banded Basslet, *Lipogramma evides* Robins & Colin, 1979, was described based on six specimens collected from Barbuda, Jamaica, Mexico, and Nicaragua. The original description also included observations of the species from Belize by Colin (1974). Subsequently, six additional specimens from the Bahamas were reported by Gilmore and Jones (1988). Robins and Colin (1979) noted differences in pigment pattern between adults and what they thought was a juvenile *Lipogramma evides* (Fig. 1A, B), in particular the presence of broader and more intense dark bands on the juvenile that completely encircle the body. Gilmore and Jones (1988) further commented on the presumed color differentiation between ontogenetic stages and noted that heavily banded “juveniles” (Fig. 1C) inhabit shallower waters (< 200 m) than adults (as deep as 250 m).

Exploratory submersible diving to 300 m in the southern and eastern Caribbean over the past several years by the Smithsonian Institution’s Deep Reef Observation Project (DROP) has resulted in the collection of over 50 specimens of “banded basslets” assignable to *Lipogramma* based on the absence of a lateral line and presence of spinous procurrent caudal-fin rays. That material includes individuals with both pigment patterns observed by previous authors and a third pigment pattern not previously described. Genetic and morphological analyses of individuals with those three pigment patterns suggested three distinct species and show that the heavily banded pattern is not an ontogenetic feature but diagnostic of a separate species. That species reaches a smaller maximum size than *Lipogramma evides* and has a shallower depth range. The other new species is similar in size and depth of occurrence to *Lipogramma evides*. Here we describe those two new species of *Lipogramma*, morphologically and genetically compare them with *Lipogramma evides*, and discuss depth distributions and evolutionary relationships of species of the genus.

## Methods and materials

**Collecting and morphology.** Basslets were collected using Substation Curaçao’s manned submersible *Curasub* (http://www.substation-curacao.com). The sub has two flexible, hydraulic arms, one of which is equipped with a quinaldine/ethanol-ejection system and the other with a suction hose. Anesthetized fish specimens were captured with the suction hose, which empties into a vented plexiglass cylinder attached to the outside of the sub. At the surface, the specimens were photographed, tissue sampled, and fixed in 10% formalin. Measurements were made weeks to months after fixation and subsequent preservation in 75% ethanol and were taken to the nearest 0.1 mm with dial calipers or an ocular micrometer fitted into a Wild stereomicroscope. Selected preserved specimens were later photographed to document preserved pigment pattern and X-rayed with a digital radiography system. Images of supraorbital pores and tooth-like structures on gill rakers were made using a Zeiss Axiocam on a Zeiss Discovery V12 SteREO microscope. Counts and measurements follow Hubbs and Lagler (1947). Specimens were cleared and stained following the protocol of Dingerkus and Uhler (1977). Symbolism for configuration of supraneural bones, anterior neural spines, and anterior dorsal pterygiophores follows Ahlstrom et al. (1976). USNM = Smithsonian Institution, National Museum of Natural History; ANSP = Academy of Natural Sciences, Philadelphia; IRCZM = Indian River Coastal Zone Museum, Harbor Branch Foundation, Fort Pierce, Florida; UF = University of Florida, Gainesville.

**Molecular analyses.** Tissue samples for 97 specimens assignable to eight species of *Lipogramma* were used for molecular analyses (Appendix 1). Tissues of *Lipogramma rosea* Gilbert, 1979 (in Robins and Colin 1979), *Lipogramma regia* Robins & Colin, 1979, and *Lipogramma flavescens* Gilmore & Jones, 1988 were not available. Tissues were stored in saturated salt-DMSO (dimethyl sulfoxide) buffer (Seutin et al. 1991). DNA extraction and cytochrome *c* oxidase subunit I (COI) DNA barcoding were performed for 96 specimens (i.e., for all available specimens except one *Lipogramma anabantoides* – Appendix 1) as outlined by Weigt et al. (2012). Four nuclear markers were amplified and sequenced—TMO-4C4, Rag1, Rhodopsin, and Histone H3—for 18 specimens of *Lipogramma*, and one or more of those genes was sequenced for an additional three specimens (Appendix 1). Primers and PCR conditions for the nuclear markers followed Lin and Hastings (2011, 2013). Sequences were assembled and aligned using *Geneious v. 9* (Biomatters, Ltd., Aukland). A neighbor-joining (NJ) network was generated for the COI data using the K2P substitution model (Kimura 1980) in the tree-builder application in *Geneious*. Mean within- and between-species K2P genetic distances were calculated from the COI data in *MEGA v. 7* (Kumar et al. 2015). Genetic distances were considered as corroborating morphology-based species delineation if the distances between species were ten or more times the intraspecific differences (Hebert et al. 2004). The alignments of COI and nuclear genes were concatenated and phylogeny was inferred using Bayesian Inference (BI) and Maximum Likelihood (ML), partitioning by gene. For the Bayesian analysis, substitution models and partitioning scheme were chosen using PartitionFinder (Lanfear et al. 2012) according to Bayesian Information Criterion scores. The chosen scheme had the following partitions and models: COI, HKY+I+G; Histone H3 plus Rhodospin, HKY+G; TMO-4C4, K80+G; Rag1, K80+G. All partitions in the ML analysis received a GTR-GAMMA substitution model. The BI phylogeny was inferred in the program *MrBayes v. 3.2* (Ronquist et al. 2012) using two Metropolis-coupled Markov Chain Monte Carlo (MCMC) runs, each with four chains. The analysis ran for 10 million generations sampling trees and parameters every 1000 generations. Burn-in, convergence and mixing were assessed using Tracer (Rambaut and Drummond 2007) and by visually inspecting consensus trees from both runs. The ML analysis was done in the program RAxML v.8.2.9 (Stamatakis, 2014), using 20 initial random searches, and topological support was assessed using 1000 bootstrap replicates. Outgroups for the phylogenetic analysis included two species of *Gramma* and several other genera from the Ovalentaria *sensu* Wainwright et al. (2012): *Acanthemblemaria* (Labrisomidae), *Helcogramma* (Tripterygiidae), *Blenniella* (Blenniidae), and *Tomicodon* (Gobiesocidae).

To corroborate the morphologically diagnosed species using our molecular data, we conducted a coalescent-based, Bayesian species-delimitation analysis (Yang and Rannala 2010, 2014). We used the computer program BP&P ver. 3.2 (Bayesian Phylogenetics and Phylogeography – Yang and Rannala [2010], Yang [2015]), which analyzes multi-locus DNA sequence alignments under the multispecies coalescent model (Rannala and Yang 2003). We used the five DNA alignments for the 21 *Lipogramma* specimens in BP&P, with each sequence in the alignments being assigned to one of eight groups *a priori*, based on the diagnostic morphological and coloration characters discussed in the ‘Morphological Comparisons’ section below. BP&P was then used to jointly infer a species tree and calculate posterior probabilities of different species-delimitation models containing either eight species, fewer than eight species (i.e. lumping multiple ‘morphological species’), or more than eight species (i.e. splitting ‘morphological species’ into multiple cryptic species).

**Depth distributions.** To evaluate depth distributions we searched FishNet2 (www.fishnet2.net) for all *Lipogramma* specimens that were identified to species and that included data on the depth of capture. For some specimens, capture depth was given as a range of possible depths, and in instances where this range was 50 m or narrower, we took the mean depth as a proxy for a point estimate of the exact depth of capture. Broader depth ranges of capture were excluded. Depth records for *Lipogramma evides* were only included for specimens whose identifications we confirmed to avoid possible confusion with one of the two new species described here. When combined with depth data from specimens from DROP collections, this search resulted in depth records for 278 identified specimens of *Lipogramma*. We also included depth records from 83 visual observations from DROP submersible dives, excluding those observations where there was uncertainty regarding identification of the three morphologically similar species (*Lipogramma evides* and the two species described here).

**Accession numbers.** GenSeq nomenclature (Chakrabarty et al. 2013) and GenBank accession numbers for DNA sequences derived in this study are presented along with museum catalog numbers for voucher specimens in Appendix 1.

## Taxonomy

### Hourglass Basslet

#### Lipogramma levinsoni

Taxon classificationAnimaliaPerciformesGrammatidae

Baldwin, Nonaka & Robertson sp. n.

http://zoobank.org/C12172C1-B3BF-48B8-B267-61D845EDCC63

Figure 2

Lipogramma evides Robins & Colin, 1979: 43, fig. 2, table 1, ANSP 134332, paratype from Jamaica (photograph, counts, measurements).
Lipogramma evides Robins & Colin, 1979, fig. 3 in Gilmore and Jones (1988: 441), IRCZM 107:07660 from San Salvador, Bahama Islands (illustration, habitat information).

##### Type locality.

Curaçao, southern Caribbean.

##### Holotype.

USNM 406139, 28.3 mm SL, tissue no. CUR11139, Curasub submersible, sta. CURASUB11-02, Curaçao, off Substation Curaçao, 12.083197 N, 68.899058 W, 137–146 m depth, 23 May 2011, C. Baldwin, D. Robertson & B. Van Bebber.

##### Paratypes.

**BONAIRE**: USNM 426784, 24.2 mm SL, tissue no. CUR13183, Curasub submersible, Bonaire, Bonaire City Dock, Kralendijk, Dive 2, 12.15 N, 68.2829 W, 121-137 m depth, 30 May 2013, B. Van Bebber, A. Schrier, C. Baldwin, T. Christiaan; **CURAÇAO**: ANSP 201863, 24.0 mm SL, Curasub submersible, Curaçao, off Substation Curaçao, 12.083197 N, 68.899058 W, no depth data available; UF 238589, 25.0 mm SL, tissue no. CUR11018, Curasub submersible, sta. CURASUB11-22, Curaçao, off of Substation Curaçao downline, 12.083197 N, 68.899058 W, no depth data available, 27 February 2011, C. Baldwin & L. Weigt; USNM 406393, 25.7 mm SL, tissue no. CUR11393, Curasub submersible, sta. CURASUB11-06, Curaçao, 132 m depth, 31 May 2011, C. Baldwin, A. Driskell, A. Schrier & B. Van Bebber; USNM 414877, 25.3 mm SL, cleared and stained, tissue no. CUR12159, Curasub submersible, sta. CURASUB12-15, Curaçao, off of Substation Curaçao downline, 12.083197 N, 68.899058 W, 128 m depth, 10 August 2012, A. Schrier, B. Brandt, C. Baldwin, A. Driskell & P. Mace; USNM 440229, 12.7 mm SL, Curasub submersible, sta. CURASUB14-07, Curaçao, in between Porto Marie and Daaibooi beaches, 12.202842 N, 69.089507 W, 123 m depth, 21 March 2014, C. Baldwin et al.; USNM 440230, 13.4 mm SL, Curasub submersible, sta. CUR13-18, Curaçao, Playa Forti, Westpoint, 12.3679 N, 69.1553 W, 127 m, 15 August 2013, C. Baldwin, B. Brandt, A. Schrier, K. Johnson & C. DeForest; USNM 406140, 19.5 mm SL, tissue no. CUR11140, Curasub submersible, sta. CURASUB11-02, Curaçao, 137-146 m depth, 23 May 2011, C. Baldwin, D. Robertson & B. Van Bebber. **DOMINICA**: USNM 440231, 17.0 mm SL, tissue no. DOM16229, Curasub submersible, off northwest Dominica, no specific collection data available, March 2016, R/V Chapman Crew.

##### Non-type specimens.

**BONAIRE**: USNM 426754, 21.2 mm SL, tissue no. CUR13184, Curasub submersible, Bonaire, Bonaire City Dock, Kralendijk, Dive 2, 12.15 N, 68.2829 W, 121–137 m depth, 30 May 2013, B. Van Bebber, A. Schrier, C. Baldwin, T. Christiaan; USNM 426802, 9.4 and 18.3 mm SL, Curasub submersible, Bonaire, Bonaire City Dock, Kralendijk, 12.15 N, 68.2829 W, 114–137 m depth, 30 May 2013, B. Van Bebber, A. Schrier, C. Baldwin, T. Christiaan. **CURAÇAO**: USNM 426774, 17.6 mm SL, tissue no. CUR13267, Curasub submersible, sta. CURASUB13-18, Curaçao, Playa Forti, Westpoint, 12.3679 N, 69.1553 W, 118 m depth, 15 August 2013, C. Baldwin, B. Brandt, A. Schrier, K. Johnson & C. DeForest; USNM 426730, 12.3 mm SL, tissue no. CUR13268, Curasub submersible, sta. CURASUB13-18, Curaçao, Playa Forti, Westpoint, 12.3679 N, 69.1553 W, 118 m depth, 15 August 2013, C. Baldwin, B. Brandt, A. Schrier, K. Johnson & C. DeForest; USNM 406011, 20.9 mm SL, tissue no. CUR11011, Curasub submersible, sta. CURASUB11-22, Curaçao, off of Substation Curaçao downline, 12.083197 N, 68.899058 W, no depth data available, 27 February 2011, C. Baldwin & L. Weigt; USNM 406012, 18.0 mm SL, tissue no. CUR11012, Curasub submersible, sta. CURASUB11-22, Curaçao, off of Substation Curaçao downline, 12.083197 N, 68.899058 W, no depth data available, 27 February 2011, C. Baldwin & L. Weigt; USNM 406019, 14.0 mm SL, tissue no. CUR11019, Curasub submersible, sta. CURASUB11-22, Curaçao, off of Substation Curaçao downline, 12.083197 N, 68.899058 W, no depth data available, 27 February 2011, C. Baldwin & L. Weigt; USNM 406394, 22.2 mm SL, tissue no. CUR11394, Curasub submersible, sta. CURASUB11-06, Curaçao, 132 m depth, 31 May 2011, C. Baldwin, A. Driskell, A. Schrier & B. Van Bebber. **DOMINICA**: USNM 438703, 19.0 mm SL, tissue no. DOM16052, Curasub submersible, sta. CURASUB16-07, Toucari Bay, Toucari, Dominica, NW corner of island, 15.608047 N, 61.471788 W, no depth data available, 2 March 2016, A. Schrier, R. Bakmeijer, B. Van Bebber & F. van der Hoeven; **JAMAICA**: ANSP 134332, 12.6 mm SL, Nekton Gamma dive 141, collection 151-2, Jamaica, Discovery Bay, 145 m depth, 15 August 1972, L. Land & S. Hastings.

##### Diagnosis.

A species of *Lipogramma* distinguishable from congeners by the following combination of characters: pectoral-fin rays 16–18 (modally 17), gill rakers 17–20 (modally 19); three supraorbital pores present along dorsal margin of orbit, no pore present between pore at mid orbit and one at posterodorsal corner of orbit; caudal fin truncate, tips of lobes rounded; body with three broad blackish bars (one on head, two on trunk) on white background, width of bar on head sufficient to encompass entire eye, width just ventral to eye averaging 26.4% head length; trunk bars sometimes hourglass shaped, with narrower and less intensely colored central regions; anterior trunk bar covering pectoral-fin base; posterior trunk bar extending onto dorsal and anal fins as large oval blotches bordered in part by white or blue pigment to form partial ocelli; dorsal and anal fins with thin orange sub-marginal stripe. The new species is further differentiated from congeners for which molecular data are available in mitochondrial COI and nuclear Histone 3, Rhodopsin, TMO-4C4, and RAG1.

##### Description.

Counts and measurements of type specimens given in Table 1. Frequency distributions of pectoral-fin rays and gill rakers on the first arch are given in Table 2. Twenty specimens examined, 9.4 to 28.3 mm SL. Dorsal-fin rays XII, 9 (last ray composite); anal-fin rays III, 8 (last ray composite); pectoral-fin rays 16–18, modally 17, 17 on both sides in holotype; pelvic-fin rays I,5; total caudal-fin rays 25 (13 + 12), principal rays 17 (9 + 8), spinous procurrent rays 6 (III + III), and 2 additional rays (i + i) between principal and procurrent rays that are neither spinous nor typically segmented; vertebrae 25 (10 + 15); pattern of supraneural bones, anterior dorsal-fin pterygiophores and dorsal-fin spines 0/0/0+2/1+1/1/; ribs on vertebrae 3–10; epineural bones present on vertebrae 1-16 in holotype and cleared and stained paratype (difficult to assess in radiographs of most other specimens); gill rakers on first arch 17–20 (5-6 + 12–14), modally 19 (6 + 13), 19 (6 + 13) in holotype; uppermost four and lowermost one or two rakers very small or present only as nubs, all other gill rakers elongate and slender with tooth-like secondary rakers as in *Lipogramma evides* (Fig. 3); pseudobranchial filaments 5–7 (7 in holotype), filaments fat and fluffy; branchiostegals 6.

**Figure 2.**
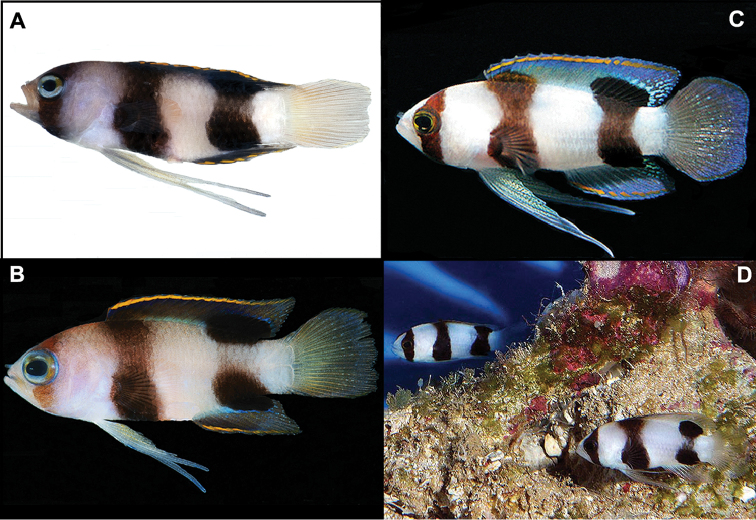
*Lipogramma levinsoni* sp. n. **A** USNM 406139, holotype, 28.3 mm SL, photographed prior to preservation, photo by D. R. Robertson and C. C. Baldwin **B** USNM 406394, 22.2 mm SL , photographed prior to preservation, photo by D. R. Robertson and C. C. Baldwin **C** and **D** Aquarium photos, Curaçao Sea Aquarium, photos by D. Ross Robertson.

**Figure 3.**
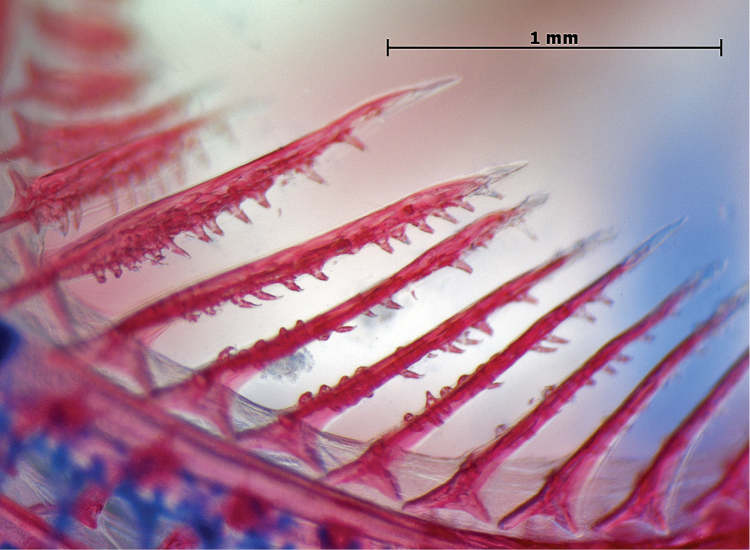
Tooth-like, secondary rakers on the first gill arch in *Lipogramma evides*, USNM 34771, cleared and stained paratype. Photo by L. Tornabene.

**Table 1.** Counts and measurements of type specimens of *Lipogramma levinsoni* sp. n.. Measurements are in percent SL except width of bar ventral to eye, which is in percent head length. C&S = cleared and stained; CP = caudal peduncle; PFO = pelvic-fin origin; P1 = pectoral fin; P2 = pelvic fin; DXII = twelfth dorsal-fin spine. “Other Caudal” rays include “i” – a slender, flexible, non-spinous, and typically non-segmented ray and “I” – a spinous procurrent ray.

	USNM 406139	USNM 406393	USNM 406140	USNM 414877	USNM 426784	USNM 440230	USNM 440229	USNM 440231	ANSP 201863	UF 238589
	Holotype	Paratype	Paratype	Paratype (C&S)	Paratype	Paratype	Paratype	Paratype	Paratype	Paratype
SL	28.3	25.7	19.5	25.3	24.2	13.4	26.3	17.0	24.0	25.0
Dorsal-fin rays	XII, 9	XII, 9	XII, 9	XII, 9	XII, 9	XII, 9	XII, 9	XII, 9	XII, 9	XII, 9
Anal-fin rays	III, 8	III, 8	III, 8	III, 8	III, 8	III, 8	III, 8	III, 8	III, 8	III, 8
Principal caudal	9+8	9+8	9+8	9+8	9+8	9+8	9+8	Broken	9+8	9+8
Other caudal	IIIi+iIII	IIIi+iIII	IIIi+iIII	IIIi+iIII	IIIi+iIII	IIIi+iIII	IIIi+iIII	Broken	IIIi+iIII	IIIi+iIII
Pectoral-fin rays	17, 17	17, 16	17, 17	17, 17	17*, 17	17, 17	17, 18	16, -	17, 17	17, 17
Gill rakers	19	20	19	19	19	19	18	18	19	18
Head length	33.2	36.2	35.4	-	38.0	37.3	34.6	39.4	35.8	37.0
Eye diameter	12.0	12.1	14.9	-	11.6	15.7	11.4	13.5	12.1	13.0
Snout length	7.1	7.4	6.2	-	7.4	6.7	5.7	5.9	7.1	5.5
Depth at CP	20.1	18.7	17.4	-	19.8	18.7	16.3	17.6	17.1	17.2
Depth at PFO	33.9	36.6	34.4	-	35.5	35.1	35.4	31.2	40.0	33.6
Length P1	25.8	24.5	23.1	-	21.9	26.1	24.7	22.4	28.8	25.6
Length P2	72.4	45.5	Broken	-	47.1	42.5	62.7	Broken	83.3	Broken
Length DXII	18.7	20.2	20.5	-	16.5	17.2	19.0	15.9	22.2	21.0
Width of bar ventral to eye	25.5	28.0	21.5	-	26.1	26.0	23.1	25.4	25.6	28.4

* 6^th^ and 7^th^ rays (counting from dorsalmost ray) separate proximally but joined distally within same sheath and appearing as a single fat ray.

**Table 2.** Frequency distributions of gill rakers on first arch and left and right pectoral-fin rays in *Lipogramma levinsoni* sp. n., *Lipogramma evides*, and *Lipogramma haberi* sp. n. Counts for the holotype and three paratypes of *Lipogramma evides* are included from Robins and Colins (1979). Counts of gill rakers and pectoral-fin rays for a fourth paratype of *Lipogramma evides*, ANSP 134330, were not given in the original description. The fifth and smallest paratype, ANSP 134332, is a specimen of *Lipogramma levinsoni*, and counts of that specimen made in this study are included. An asterisk indicates count of gill rakers or left pectoral-fin rays in holotype.

	Gill Rakers								Pectoral-fin Rays				
	15	16	17	18	19	20	21	22	15	16	17	18	19
*Lipogramma levinsoni*			1	5	9*	1				5	26*	3	
*Lipogramma evides*					3	14*	11	1	1	45*	9		
*Lipogramma haberi*	1*	2							1	5*			

Spinous and soft dorsal fins confluent, several soft rays at rear of fin forming elevated lobe that extends posteriorly beyond base of caudal fin. Pelvic fin, when depressed, extending posteriorly to point between anterior base of anal fin and beyond base of caudal fin, elongate first pelvic-fin ray broken in most preserved specimens. Dorsal profile from snout to origin of dorsal fin convex. Diameter of eye of holotype contained 2.8 times in head length. Pupil slightly tear shaped, with small aphakic space anteriorly. Scales extending anteriorly onto posterior portion of head, ending short of coronal pore. Scales present on cheeks, opercle, preopercle, interopercle, and isthmus. Scales lacking on top of head, snout, jaws, and branchiostegals. Scales large and deciduous, too many scales missing in most specimens to make accurate scale counts. In holotype, approximately 23 lateral scales between shoulder and base of caudal fin, approximately 4 scale rows on cheek, and approximately 9 scale rows across body above anal-fin origin. Scales on head and nape without cteni, scales on rest of body ctenoid. Fins naked except small scales present at bases of soft dorsal and anal fins.

**Figure 4.**
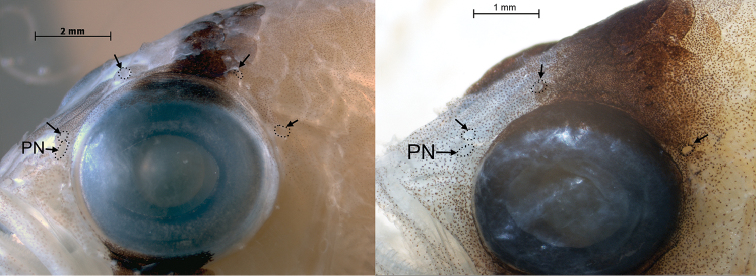
Supraorbital pore patterns in *Lipogramma evides*, UF 238591, 34.5 mm SL (left) and *Lipogramma levinsoni* sp. n., UF 238589, 25.0 mm SL (right). Arrows point to pores, which have been outlined with tiny dots for emphasis. PN – posterior nostril.

Margins of bones of opercular series smooth, opercle without spines. Single row of teeth on premaxilla posteriorly, broadening to 2-3 rows anteriorly, teeth in innermost row smallest, some teeth in outer row enlarged into small canines. Dentary similar, holotype with 3 enlarged teeth in outer row near symphysis. Vomer with chevron-shaped patch of teeth, palatine with long series of small teeth. Several canals and pores visible on head, but most pores inconspicuous. Conspicuous pores present in infraorbital canal (2 pores) and portion of supraorbital canal bordering dorsal portion of orbit (3); less conspicuous pores present on top of head (1 median coronal pore), preopercle (7), and lateral-line canal in the posttemporal region (3). Anteriormost of the 3 supraorbital pores situated at anterodorsal corner of orbit, middle supraorbital pore situated above mid orbit, and posteriormost supraorbital pore situated at posterodorsal corner of orbit (Fig. 4). This pore with fleshy rim in holotype, and mid-orbit supraorbital pore with smaller fleshy rim. Posterior nostril situated just ventral to anteriormost supraorbital pore, nostril a single large opening with ventral portion of rim slightly elevated. Anterior nostril in tube with anterior flap and situated just posterior to upper lip. No lateral line present on body.

**Figure 5.**
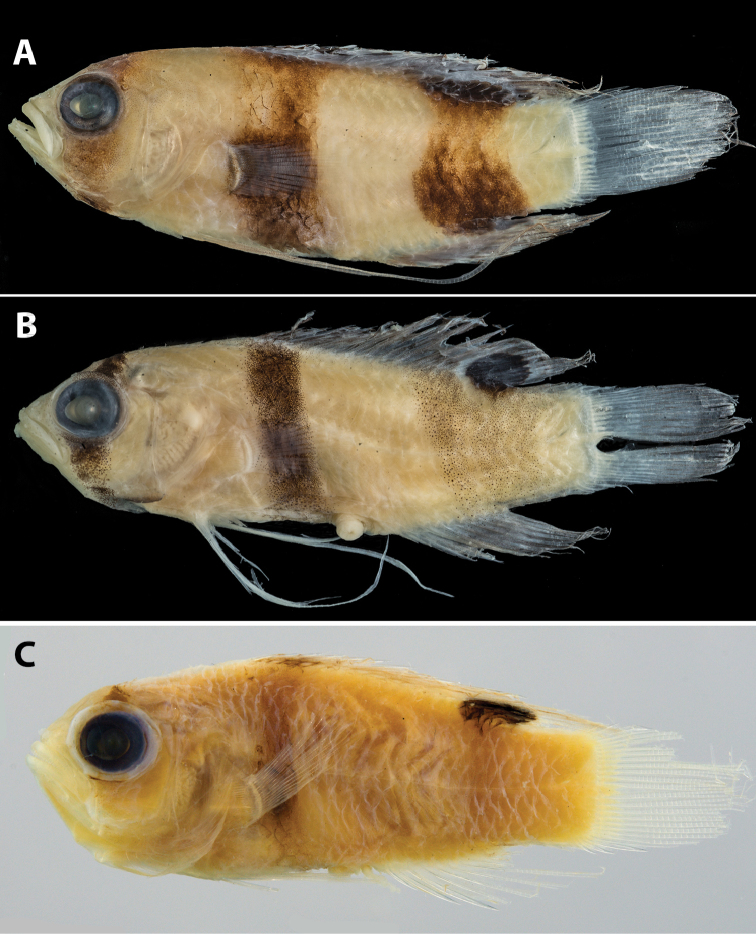
Preserved specimens of **A** *Lipogramma levinsoni* sp. n., holotype, USNM 406139, 28.3 mm SL **B** *Lipogramma haberi* sp. n., holotype, USNM 422679, 40.1 mm SL **C** *Lipogramma evides*, paratype, ANSP 134330, 30.5 mm SL Photos **A** and **B** by Sandra Raredon, **C** by Mark Sabaj.

Coloration: In life (Fig. 2), ground color of head and trunk white to tan dorsally grading to white below. **Head**: dark brown to black bar encompassing orbit and extending ventrally to ventral midline; above orbit, bar narrowing across dorsal midline; eye with dark brown outer ring, yellowish to bluish iris. **Trunk**: two broad, dark brown to blackish bars present beneath dorsal fin, bars sometimes hourglass shaped, with narrower and less intensely colored central regions (central regions losing almost all dark color in some freshly dead specimens); anterior bar extending ventrally from anterior third of spinous dorsal fin to ventral midline, its anterior border extending forward to encompass base of pectoral fin; posterior bar extending ventrally from base of soft dorsal fin to posterior half of anal fin. **Dorsal fin**: dark trunk bars extending onto base of fin as two blotches, anterior blotch short, low, less conspicuous (than posterior blotch) and sometimes with faint orange upper border. Posterior blotch an intense, dark, longitudinal oval spanning lower half of soft dorsal and bordered posteriorly by white to bluish-white pigment. Base of fin between trunk bars whitish, central portion of fin brown to grey, and distal third of fin with bluish tint and thin, orange, submarginal stripe; this stripe breaking into spots along the rear third of fin. **Anal fin**: posterior trunk bar extending onto proximal portion of posterior half of fin as a strong, horizontally elongate, black blotch edged distally with bluish white line; base of fin with thin, white stripe, fin color grading into blackish to bluish-black distally. A thin, orange, sub-marginal stripe breaks into spots along posterior portion of fin. **Caudal fin**: basal half translucent pale orange, grading into translucent bluish distally, sometimes with indistinct, very narrow, submarginal orange band around entire edge. **Pectoral fins**: base blackish, fin translucent, rays translucent or tinted with orange. **Pelvic fins**: translucent white to bluish white, with orange tint medially on basal half of fin. In preservative (Fig. 5A), barred color pattern retained, but orange, yellow, and bluish pigments absent.

##### Distribution.

Known from specimens collected from the Bahamas, Bonaire, Curaçao, Dominica, and Jamaica. This species was also clearly observed in October 2016 by DRR and LT from the mini-submarine “Idabel” at 140 m depth adjacent to Half Moon Bay, Roatan, Honduras.

##### Habitat.

Lives in or hovers above small rocky rubble on gradual slopes at depths of 108-154 m. When approached by the submersible, *Lipogramma levinsoni* disappears into the rubble. We observed them often in pairs.

##### Etymology.

Named *Lipogramma levinsoni* in recognition of the generous, continuing support of research on neotropical biology at the Smithsonian Tropical Research Institute (Panamá) made by Frank Levinson.

##### Common name.

We propose “Hourglass basslet” (Cabrilleta hierba-horaria as the Spanish equivalent) to differentiate this species from the Banded Basslet, *Lipogramma evides*, and the Yellow-banded Basslet, *Lipogramma haberi* (see description below), both of which have narrower, straight-sided bars on the trunk.

##### Genetic comparisons.

Table 3 shows average inter- and intraspecific divergences in COI among species of *Lipogramma* analyzed genetically in this study. With the exception of a single substitution in one specimen, the 15 specimens of *Lipogramma levinsoni* exhibit no intraspecific genetic variation at this locus and differ from other *Lipogramma* species by 15.4–26.0%. *Lipogramma levinsoni* differs from *Lipogramma evides* by 17.1% and *Lipogramma haberi* by 19.0%.

**Table 3.** Average Kimura two-parameter distance summary for species of *Lipogramma* based on cytochrome c oxidase I (COI) sequences analyzed in this study. Intraspecific averages are in bold.

	“*robinsi1*”	“*robinsi2*”	*levinsoni*	*haberi*	*anabantoides*	*trilineata*	*klayi*	*evides*
“*robinsi1*” (n=6)	**0.003**							
“*robinsi2*” (n=7)	0.119	**0.002**						
*levinsoni* (n=15)	0.162	0.169	**0**					
*haberi* (n=3)	0.111	0.132	0.19	**0.002**				
*anabantoides* (n=2)	0.195	0.184	0.154	0.202	**0.005**			
*trilineata* (n=12)	0.217	0.251	0.227	0.236	0.258	**0.005**		
*klayi* (n=21)	0.266	0.259	0.26	0.279	0.246	0.242	**0.003**	
*evides* (n=30)	0.103	0.128	0.171	0.11	0.22	0.249	0.263	**0.001**

##### Comments.

**Figure 6.**
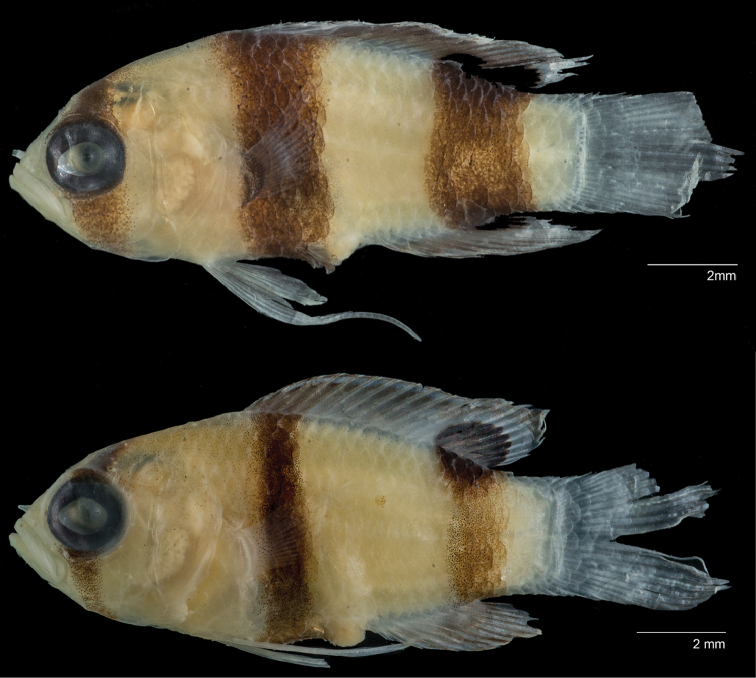
Comparison of juveniles of **A** *Lipogramma levinsoni* sp. n., USNM 440230, paratype, 13.4 mm SL and **B** *Lipogramma evides*, USNM 431410, 12.7 mm SL.

The smallest paratype of *Lipogramma evides*, ANSP 134332 (Fig. 1B), 12.6 mm SL, is a specimen of *Lipogramma levinsoni*. Although Robins and Colin (1979) indicated 15 pectoral-fin rays on both sides of this specimen, we count 17 on the right and find the left side too bent to make an accurate count. *Lipogramma levinsoni* typically has 17–18 pectoral-fin rays, modally 17. The gill-raker count of 19 given by Robins and Colin (1979) was confirmed by our examination, and is the typical count for *Lipogramma levinsoni*. Counts of pectoral-fin rays (15–16, usually 16) and gill rakers on the first arch (19–21, usually 20 or 21) given by Robins and Colin (1979) for the remaining paratypes of *Lipogramma evides* support their identification as specimens of *Lipogramma evides*. As noted, previous authors have mistakenly identified the broad-banded *Lipogramma levinsoni* as the juvenile form of the more narrow-banded *Lipogramma evides*. Our material includes juvenile specimens of both *Lipogramma levinsoni* and *Lipogramma evides*, which in each case have the adult configuration of dark bands (Fig. 6).

### Yellow-banded Basslet

#### Lipogramma haberi

Taxon classificationAnimaliaPerciformesGrammatidae

Baldwin, Nonaka & Robertson sp. n.

http://zoobank.org/4A8447E9-205C-4639-9209-428D8DCDAC1F

Figure 7

##### Type locality.

Curaçao, southern Caribbean

##### Holotype.

USNM 422679, 40.1 mm SL, tissue no. CUR13171, Curasub submersible, sta. CURASUB13-09, Curaçao, southwest tip of Klein Curaçao, 11.975783 N, 68.646192 W, 152 m depth, 27 May 2013, M. Harasewych, L. Weigt, B. Van Bebber & A. Schrier.

##### Paratypes.

USNM 434772, 26.4 mm SL, tissue no. CUR15092, Curasub submersible, sta. CURASUB15-12, northwest corner of Klein Curaçao, 11.998453 N, 68.651308 W, 187 m depth, 27 August 2015, B. Brandt, A. Schrier, S. Haber & T. Haber; USNM 422670, 23.0 mm SL, tissue no. CUR13158, Curasub submersible, sta. CURASUB13-08, Curaçao, southwest tip of Klein Curaçao, 11.975783 N, 68.646192 W, 233 m depth, 27 May 2013, C. Baldwin, D. Robertson, B. Brandt, A. Schrier & L. Weigt.

##### Diagnosis.

A species of *Lipogramma* distinguishable from congeners by the following combination of characters: pectoral-fin rays 15–16 (modally 16), gill rakers 15–16 (modally 16); four supraorbital pores along dorsal portion of orbit, a pore present between pore at mid orbit and one at posterodorsal corner or orbit; caudal fin truncate, tips of lobes rounded; body with three dusky bars (one on head, two on trunk) on yellow/white background; width of bar on head sufficient to encompass pupil but not entire eye, width just ventral to eye averaging 17.6% head length; anterior trunk bar narrow and not extending forward to cover pectoral-fin base, bar lighter and less conspicuous ventrally; posterior trunk bar a broad, yellow/tan triangle that is wider dorsally than ventrally; this triangle extending onto soft dorsal fin as large, round, well-defined ocellus; posterior trunk bar not extending onto anal fin; dorsal fin with thin yellow sub-marginal stripe; no yellow submarginal stripe on anal fin; dorsal, anal, and caudal fins with numerous yellow spots. The new species is further differentiated from congeners for which molecular data are available in COI and RAG1.

##### Description.

Counts and measurements of type specimens given in Table 4. Frequency distributions of pectoral-fin rays and gill rakers on the first arch are given in Table 2. Three specimens examined, 23.0–40.1 mm SL. Dorsal-fin rays XII, 9 (last ray composite); anal-fin rays III, 8 (last ray composite); pectoral-fin rays 15–16, modally 16, 16 on both sides in holotype; pelvic-fin rays I,5; total caudal-fin rays 25 (13 + 12), principal rays 17 (9 + 8), spinous procurrent rays 6 (III + III), and 2 additional rays (i + i) between principal and procurrent rays that are neither spinous nor typically segmented; vertebrae 25 (10 + 15); pattern of supraneural bones, anterior dorsal-fin pterygiophores and dorsal-fin spines 0/0/0+2/1+1/1/; ribs on vertebrae 3-10; epineural bones present on vertebrae 1–15 in one paratype, difficult to assess in other specimens; gill rakers on first arch 15–16 (4-5 + 11), 15 (4 + 11) in holotype, both paratypes with 16 (5 + 11); lowermost two rakers very small, all other gill rakers elongate and slender with tooth-like secondary rakers as in *Lipogramma evides* (Fig. 3); pseudobranchial filaments 6, filaments fat and fluffy; branchiostegals 6.

**Table 4.** Counts and measurements of type specimens of *Lipogramma haberi* sp. n. Measurements are in percent SL except width of bar ventral to eye, which is in percent head length. CP = caudal peduncle; PFO = pelvic-fin origin; P1 = pectoral fin; P2 = pelvic fin; DXII = twelfth dorsal-fin spine. “Other Caudal” rays include “i” – a slender, flexible, non-spinous, and typically non-segmented ray and “I” – a spinous procurrent ray.

	USNM 422679	USNM 434772	USNM 422670
	Holotype	Paratype	Paratype
SL	40.1	26.4	23.0
Dorsal-fin Rays	XII, 9	XII, 9	XII, 9
Anal-fin Rays	III, 8	III, 8	III, 8
Principal Caudal	9+8	9+8	Broken
Other Caudal	IIIi+iIII	IIIi+iIII	Broken
Pectoral-fin Rays	16, 16	16, 15	16, 16
Gill Rakers	15	16	16
Head Length	35.2	39.0	34.8
Eye Diameter	11.2	14.0	13.0
Snout Length	6.7	5.7	6.1
Depth at CP	18.7	20.1	17.8
Depth at PFO	32.4	34.1	27.0
Length P1 Fin	22.2	27.7	24.3
Length P2 Fin	62.3	54.5	46.1
Length DXII	22.4	23.1	17.4
Width of Bar Ventral to Eye	14.9	20.4	17.5

Spinous and soft dorsal fins confluent, several soft rays in posterior portion of fin forming elevated lobe that extends posteriorly beyond base of caudal fin. Pelvic fin extending posteriorly to anterior third of caudal peduncle in holotype when depressed, longest pelvic-fin rays broken in preserved specimens. Dorsal profile from snout to origin of dorsal fin convex. Diameter of eye of holotype contained 2.7 times in head length. Pupil slightly tear shaped, with small aphakic space anteriorly. Scales extending anteriorly onto top of head, ending short of coronal pore. Scales present on cheeks, opercle, preopercle, interopercle, and isthmus. Scales lacking on frontal region, snout, jaws, and branchiostegals. Scales large and deciduous, too many missing in paratypes to make counts, holotype with approximately 24 lateral scales between shoulder and base of caudal fin, 5 cheek rows, and 11 rows across body above anal-fin origin. Scales on head and nape without cteni, scales on rest of body ctenoid. Fins naked except small scales present at bases of soft dorsal and anal fins.

Margins of bones of opercular series smooth, opercle without spines. Premaxilla with band of small conical teeth, band widest at symphysis, outer row with largest teeth, 3 or 4 near symphysis enlarged into canines. Dentary similar except 4-6 anterior teeth enlarged into canines. Vomer with chevron-shaped patch of teeth, palatine with long series of small teeth. Several canals and pores visible on head, but most pores inconspicuous. Conspicuous pores present in infraorbital canal (2) and in supraorbital canal bordering dorsal portion of orbit (4); less conspicuous pores present on top of head (1 median coronal pore), preopercle (8), and lateral-line canal in posttemporal region (3). An additional 4 tiny pores present beneath orbit in holotype in infraorbital canal. Supraorbital pore pattern as in *Lipogramma evides* (Fig. 4): anteriormost of 4 supraorbital pores situated at anterodorsal corner of orbit, second supraorbital pore situated above mid orbit, and posteriormost supraorbital pore situated at posterodorsal corner of orbit. Between second and posteriormost supraorbital pores, another pore present and situated closer to latter. Posterior nostril situated just ventral to anteriormost supraorbital pore, nostril a single large opening with ventral portion of rim slightly elevated. Anterior nostril in tube with anterior flap and situated just posterior to upper lip. No lateral line present on body.

**Figure 7.**
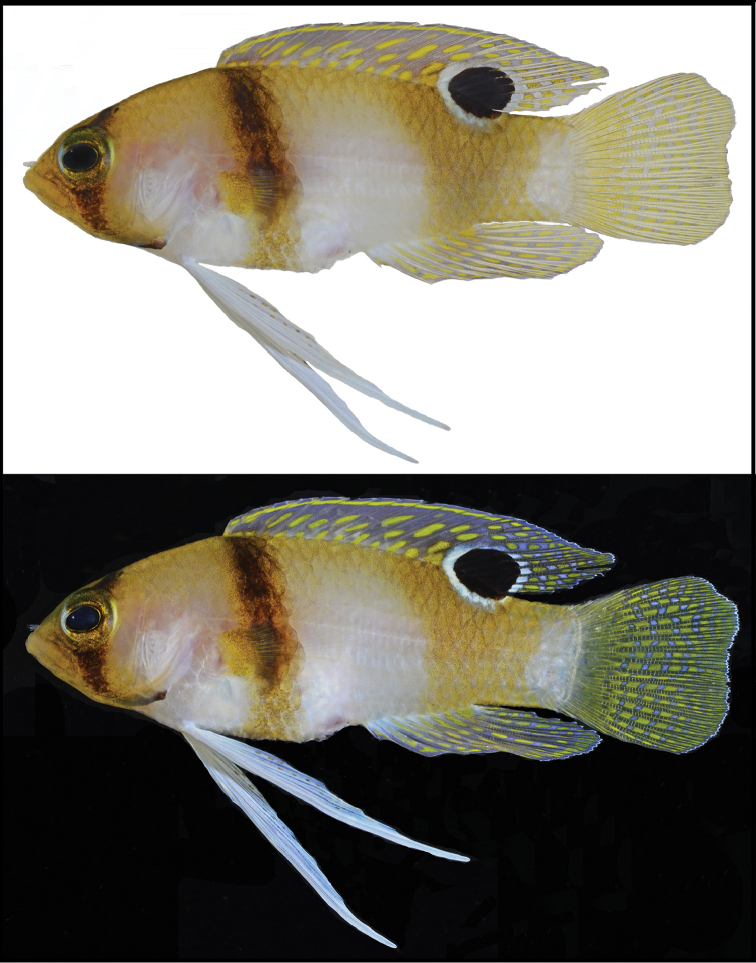
*Lipogramma haberi* sp. n., USNM 422679, holotype, 40.1 mm SL, photographed prior to preservation against white (top) and black (bottom) backgrounds. Photos by D. R. Robertson and C. C. Baldwin.

Coloration: In life, ground color of head and trunk pale yellow to tan dorsally, white ventrally. **Head**: mostly pale yellow-tan with white blotch on operculum; a brown to black C-shaped bar with yellow-brown edges originating on top of head, widening ventrally above orbit to width of pupil and passing over orbit at that width, then narrowing ventrally and continuing as dark line along lower edge of operculum; iris dark brown above and below where bar passes through, yellowish-white anteriorly and posteriorly, a thin gold ring circling pupil. **Trunk**: two dark bars beneath dorsal fin, anterior one brown to blackish (edged with yellow-brown) originating below anterior dorsal spines and descending obliquely behind pectoral-fin base to ventral midline; bar fading below pectoral-fin base; posterior bar much broader than anterior bar but paler and less conspicuous, bar spanning dorsal and ventral body margins and covering anterior half of caudal peduncle; bar narrowing ventrally. **Dorsal fin**: grey with a bluish tint (when photographed against black background – Fig. 7, bottom), with thin, submarginal yellow stripe; spinous dorsal fin with row of round to oblong yellow spots along base, 1–2 rows of obliquely oriented, oval, yellow spots above that; soft dorsal with large, conspicuous, circular, black ocellus covering lower half of fin and extending onto dorsal portion of trunk; thin, white, outer ring surrounding ocellus on both fin and trunk complete in holotype (Fig. 7), absent along underside of ocellus in both paratypes; above ocellus, soft dorsal fin with approximately three rows of rounded yellow spots; grey spaces between yellow spots appearing as well-defined grey to blue spots posteriorly. **Anal fin**: grey with bluish tint (when photographed against black background), each ray with 3-6 elongate yellow spots from base to fin edge; grey spaces between yellow spots appearing as well-defined grey to blue spots posteriorly. **Caudal fin**: base of fin mostly yellow, remainder of fin with rows of yellow spots along fin rays; grey spaces between yellow spots appearing as well-defined grey or blue spots. **Pectoral fins**: base yellowish with black dots, fin translucent. **Pelvic fins**: bright white, inner 2-3 rays with series of small yellow-brown dots. In preservative (Fig. 5B), barred color pattern retained, posterior trunk bar faint, and yellow and bluish pigments absent.

##### Distribution.

Known only from Klein Curaçao, a 1.7 km^2^ island 11 km southeast of Curaçao.

##### Habitat.

No specific habitat information recorded.

##### Etymology.

Named in honor of Spencer and Tomoko Haber, who funded and participated in a submersible dive by the Smithsonian’s Deep Reef Observation Project (DROP) that resulted in the collection of USNM 434772, a paratype of the new species.

##### Common name.

We propose “Yellow Banded Basslet” (“Cabrilleta cinta-amarilla” as the spanish equivalent) to distinguish *Lipogramma haberi* from *Lipogramma evides* and *Lipogramma levinsoni*. Although *Lipogramma evides* has a submarginal yellow stripe along the dorsal and anal fins, it lacks the overall yellow body color of *Lipogramma haberi*.

##### Genetic comparisons.

Table 3 shows average inter- and intraspecific divergences in COI among species of *Lipogramma* analyzed genetically in this study. *Lipogramma haberi* exhibits 0.2% intraspecific genetic variation and 11.0–27.9% divergence from other *Lipogramma* species. It differs from *Lipogramma evides* by 11.0% and from *levinsoni* by 19.0%.

##### Comments.

Relative to *Lipogramma levinsoni* and *Lipogramma evides*, which are known from multiple localities within the Caribbean Sea, *Lipogramma haberi* is an uncommon species on deep reefs and may have a more restricted geographic distribution. Although both *Lipogramma levinsoni* and *Lipogramma evides* are frequently observed and collected off the southern coast of Curaçao, in more than one hundred submersible dives there we have not collected *Lipogramma haberi*. Rather, we have only collected *Lipogramma haberi* on infrequent trips to Klein Curaçao, a small island, as noted above, 11 km southeast of Curaçao.

## Discussion

**Figure 8.**
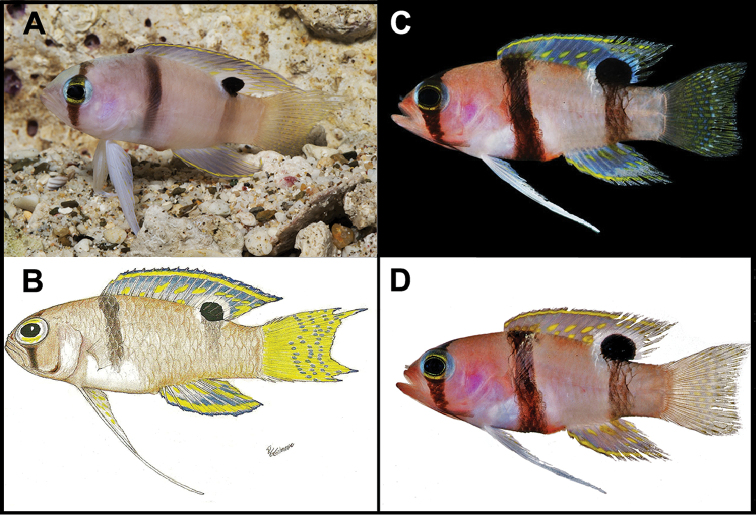
*Lipogramma evides* **A** Aquarium photograph by Barry Brown, Substation Curacao **B** USNM 276560, 45.3 mm SL, illustration by Grant Gilmore in Gilmore and Jones (1988: fig. 1) **C** and **D** USNM 414885, 24.4 mm SL, photos by D. R. Robertson and C. C. Baldwin against black (**C**) and white (**D**) backgrounds.

**Comments on *Lipogramma evides*.** The type series of *Lipogramma evides* includes the holotype and five paratypes (Robins and Colin 1979). We examined specimens or photographs of specimens of the type series from ANSP and FMNH and conclude that all except one, ANSP 134332, 12.6 mm SL, represent *Lipogramma evides*. We also examined 31 specimens of *Lipogramma evides* that we recently collected at Curaçao and that range in size from 12.7–45.4 mm SL. Frequency distributions of pectoral-fin rays and gill rakers on the first arch are given in Table 2, an illustration of the holotype that was included in the original description of the species is shown in Fig. 1A, color patterns of live and recently deceased individuals are shown in Fig. 8, a photographed of a preserved paratype (ANSP 134330) is provided in Fig. 5C, secondary spines on gill rakers of the first arch are shown in Fig. 3, supraorbital pore pattern is shown in Fig. 4, and a photograph of a preserved juvenile is featured in Fig. 6.

The illustration of the holotype (Fig. 1A) shows a triangular-shaped bar on the posterior portion of the trunk that more closely resembles the shape of that bar in *Lipogramma haberi* (Fig. 7) than *Lipogramma evides* (Fig. 8). The pigment is too faded in the preserved holotype now to determine the shape of that bar, but we note that in specimens or photographs of the four paratypes of the type series that are actually specimens of *Lipogramma evides*, the posterior trunk bar is narrow (e.g., Fig. 5C). This suggests that the shape of the posterior trunk bar illustrated in the holotype of *Lipogramma evides* is either incorrectly drawn or represents an anomaly for the species. *Lipogramma evides* and *Lipogramma haberi* are easily distinguished by numbers of gill rakers on the first arch—15 or 16 in *Lipogramma haberi*, 19-22 (modally 20) in *Lipogramma evides* (Table 2). Our examination of the holotype confirms the count by Robins and Colin (1979) of 20 gill rakers in the holotype of *Lipogramma evides*. Furthermore, the triangular-shaped posterior trunk bar in *Lipogramma haberi* is very pale relative to the anterior trunk and head bars. Robins and Colin’s (1979) illustration of the holotype shows three body bars of equal intensity.

Colin’s (1974) observation of “*Lipogramma* sp.” at Glover’s Reef, Belize, was cited as *Lipogramma evides* by Robins and Colin (1979), and based on the recorded depths of observations, 165–180 m, we tentatively agree with this identification, as Belize is between Arrowsmith Bank and Nicaragua, where *Lipogramma evides* does occur. *Lipogramma evides* inhabits depths of 133-302 m, whereas *Lipogramma levinsoni* occurs from 103 to 154 m. However, Colin’s (1974) observed fish could have been *Lipogramma haberi*, which occurs from 152–233 m. Polanco et al. (2012) recorded several specimens of *Lipogramma evides* from the Coralinos Archipelago off Colombia, and based on the stated counts of 15–16 pectoral-fin rays and 20–21 gill rakers, those specimens are correctly identified. In addition to Colombia and the tentative Belize location, *Lipogramma evides* is known definitively from the type locality of Arrowsmith Bank in the Yucatan Peninsula, Nicaragua, southeast of Barbuda, and Curaçao (including Klein Curaçao). It was also observed but not collected in October 2016 by DRR and LT from the mini-submarine “Idabel” at 232–250 m depth adjacent to Half Moon Bay, Roatan, Honduras. It was not collected or observed on DROP submersible dives at Bonaire or Dominica. A list of *Lipogramma evides* material examined in this study is given in Appendix 2.

**Morphological comparisons.** *Lipogramma levinsoni*, *Lipogramma evides*, and *Lipogramma haberi* can be readily distinguished from all congeners in having three dark bars (one on the head, two on the trunk) on a white background vs. a brown body with a reddish head in *Lipogramma anabantoides*; a yellow body with one black bar on the head in *Lipogramma flavescens*; a purple head and yellow trunk in *Lipogramma klayi* Randall, 1963; a brown body with one broad white bar and multiple narrow orange bars in *Lipogramma regia*; a brownish body with about 12 thin dark bars in *Lipogramma robinsi* Gilmore, 1997; a pink head and trunk with a yellow stripe along the dorsal profile of the head in *Lipogramma rosea*; and a yellow head and trunk with three long iridescent blue stripes on the head in *Lipogramma trilineata* Randall, 1963. The major differences among *Lipogramma levinsoni*, *Lipogramma evides*, and *Lipogramma haberi* are summarized in Table 5. *Lipogramma evides* and *Lipogramma haberi* are morphologically similar and reach a similar maximum size (45.5 and 40.1 in our material, respectively). They are easy to distinguish from one another on the basis of number of gill rakers on the first arch (usually 20-21 in *Lipogramma evides*, 15-16 in *Lipogramma haberi* – Table 2) and by live and preserved color pattern (Figs 5, 7, 8). In life, *Lipogramma haberi* has a considerable amount of yellow as ground color and associated with the dark bars, whereas the ground color of *Lipogramma evides* is mostly white. In fresh and preserved specimens, the posterior trunk bar in *Lipogramma haberi* is broad and much wider at the top than the bottom, whereas in *Lipogramma evides* it is narrower and of uniform width. There is also a difference in the shape of the caudal fin of the two species, with *Lipogramma haberi* having a truncate fin with rounded lobe tips and *Lipogramma evides* having a slightly emarginate fin with pointed lobe tips.

**Table 5.** Comparisons among *Lipogramma levinsoni* sp. n., *Lipogramma haberi* sp. n., and *Lipogramma evides*.

	*Lipogramma levinsoni*	*Lipogramma haberi*	*Lipogramma evides*
Standard length (mm)	9.4–28.3	23.0–40.1	13.7–45.5
Gill rakers on 1^st^ arch	Usually 19	15–16	Usually 20–21
Pectoral-fin rays	Usually 17	Usually 16	Usually 16
Supraorbital pores/pore present between pore at mid orbit and one at posterodorsal corner of orbit (Fig. 4)	3/Absent	4/Present	4/Present
Ground color	White, grey on nape & snout	Yellow above, white below	White
Dark bar on head	Black; relatively wide, widens to encompasses entire eye No rearward extension along lower edge of opercle	Brown, edged with yellow; relatively narrow, widens to encompass pupil Narrow rearward extension along lower edge of opercle	Black; relatively narrow, widens to encompass pupil Narrow rearward extension along lower edge of opercle
Width of dark bar on head (measured immediately ventral to eye) in % HL)	21.5–34.8 (x = 26.4)	14.9–20.4 (x = 17.6)	8.7–19.4 (x = 14.7)
Anterior trunk bar	Black, wide, vertical, center often narrower & paler Covers pectoral base Extension onto dorsal fin large, intense	Brown, edged with yellow; narrow, slightly oblique, uniform width, paler on belly Behind pectoral base No extension onto dorsal fin	Black; narrow, slightly oblique, uniform width, paler on belly Behind pectoral base Extension onto dorsal fin small, weak
Posterior trunk bar	Same form and color as anterior bar Extension onto dorsal fin = oval partial ocellus Extension onto anal fin = elongate, partial ocellus	Yellow-brown; broad dorsally, thinning ventrally, triangular in shape Extension onto dorsal fin = round, well defined ocellus No extension onto anal fin	Same form and color as anterior bar but usually paler than anterior bar Extension onto dorsal fin = round, well defined ocellus Extension onto anal fin absent or a small, weak smudge
Dorsal-fin pigment	Submarginal stripe orange Remainder of fin without pale spots	Submarginal stripe yellow Remainder of fin with 2–3 rows of yellow spots	Submarginal stripe yellow Remainder of fin with 1–2 rows of yellow spots
Anal-fin pigment	Submarginal stripe orange No pale spots on remainder of fin	No pale submarginal stripe Remainder of fin with 1–6 rows of yellow spots	Submarginal stripe yellow Remainder of fin with 1–3 rows of yellow spots near base
Caudal-fin shape	Truncate, lobe tips rounded	Truncate, lobe tips rounded	Slightly emarginate, lobe tips pointed
Depth range (m)	108–154	152–233	133–302
Geographical distribution	Bahamas, Bonaire, Curaçao, Dominica, and Jamaica	Klein Curaçao	Barbuda, Belize(?),Colombia, Curaçao, Klein Curaçao, Mexico (Caribbean), and Nicaragua

*Lipogramma levinsoni* reaches a smaller maximum size than *Lipogramma haberi* and *Lipogramma evides* (largest specimen examined 28.3 mm SL) and differs in modal numbers of gill rakers on first arch and pectoral-fin rays (Table 2), supraorbital pore pattern (Fig. 4), and numerous aspects of color pattern (Figs 2, 5, 7, 8). In life, *Lipogramma levinsoni* is easily distinguished from *Lipogramma haberi* and *Lipogramma evides* by having an orange submarginal stripe on the dorsal fin (vs. yellow) and an orange submarginal stripe on the anal fin (vs. no stripe in *Lipogramma haberi*, a yellow submarginal stripe in *Lipogramma evides*). In preservative, *Lipogramma levinsoni* is easily distinguished from *Lipogramma haberi* and *Lipogramma evides* by the shape, size, and configuration of the dark body bars (Table 5).

**Species delimitation and phylogeny.** The neighbor-joining network (Suppl. material 1) shows eight distinct genetic lineages with an average within-lineage genetic distance of 0.002 substitutions/site and an average between-lineage genetic distance of 0.20 substitutions/site (Table 3). Considering between-lineage distances that are 10 or more times greater than within-lineage distances as indicative of distinct species (Hebert et al. 2014), genetic distances corroborate the recognition of the *Lipogramma levinsoni* and *Lipogramma haberi* lineages as species. Average between-lineage divergence for *Lipogramma levinsoni* is 19% (18% between *Lipogramma levinsoni* and the other two banded basslets, *Lipogramma haberi* and *Lipogramma evides*), whereas average within-lineage divergence is 0%. For *Lipogramma haberi*, average between-lineage divergence is 18% (11% between *Lipogramma haberi* and *Lipogramma levinsoni*/*Lipogramma evides*), whereas average within-lineage divergence is 0.2%.

**Figure 9.**
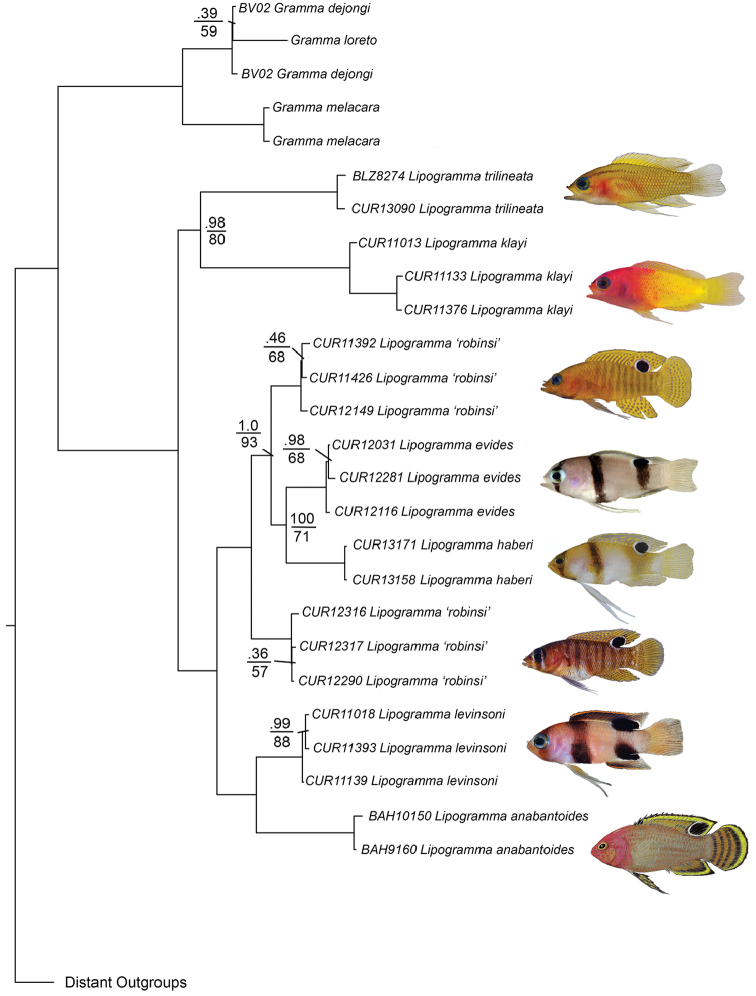
Bayesian Inference molecular phylogeny of *Lipogramma* based on combined mitochondrial and nuclear genes. Numbers of individuals analyzed for each species are given in Appendix 1, along with the genes sequenced for each individual. Topology is identical to that from Maximum Likelihood analysis. Support values are Bayesian posterior probabilities (above) and bootstrap values (below). Nodes without labels have 1.0 posterior probability and 100 bootstrap values. Photos or illustrations by C. C. Baldwin, D. R. Robertson, R. G. Gilmore, and C. R. Robins.

**Figure 10.**
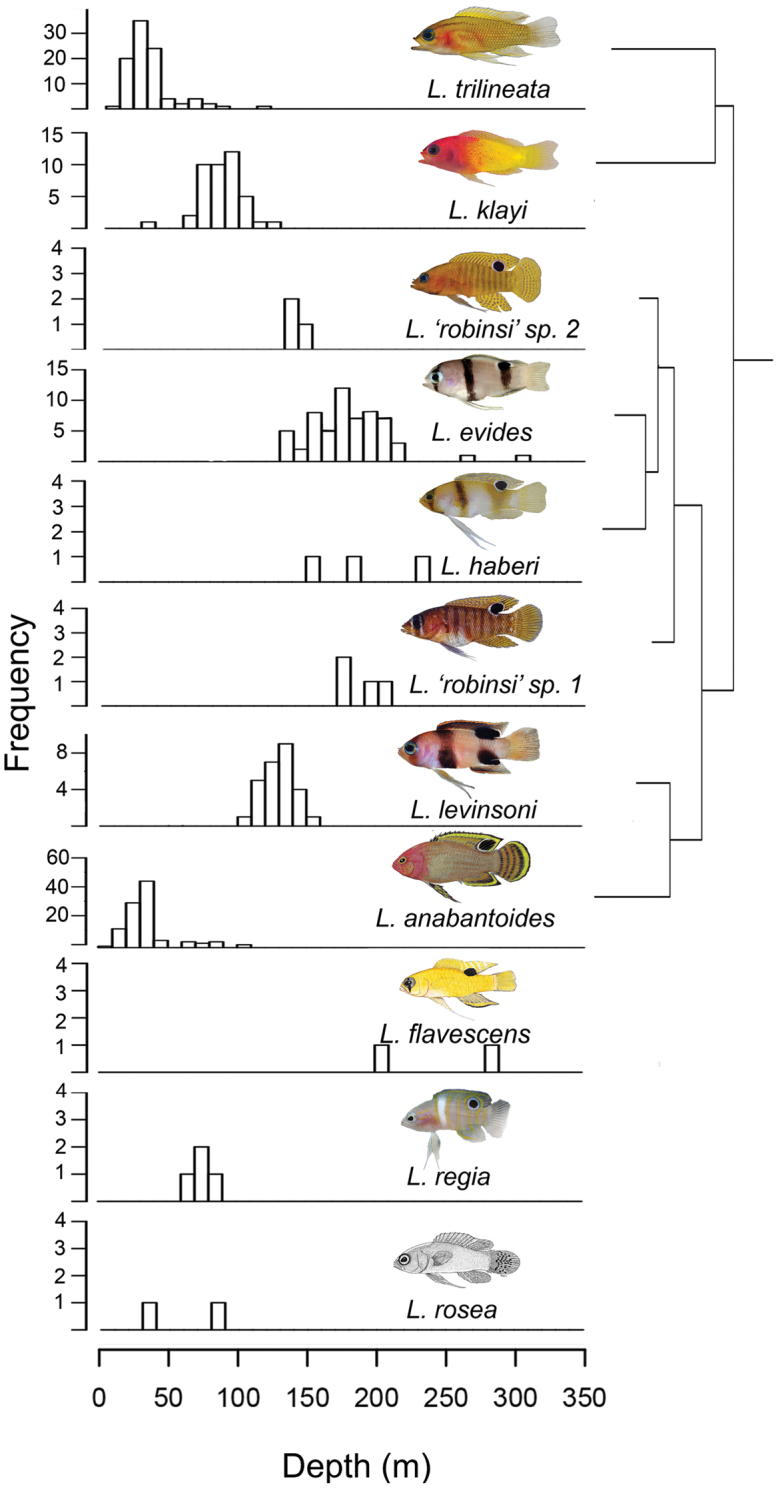
Depth distributions for species of *Lipogramma*. Photos or illustrations by C. C. Baldwin, D. R. Robertson, R. G. Gilmore, and Mooi and Gill (2002: fig. 9).

The ML and BI analyses resulted in identical topologies, with most relationships supported by 1.0 posterior probability and 100% bootstrap support (Fig. 9). The BP&P analysis inferred a coalescent-based species-tree that was identical in topology to the ML and BI trees. In addition, the BP&P analysis provided overwhelming support for the presence of eight species of *Lipogramma* in our phylogeny (posterior probability 0.99981), including three distinct species of banded basslets (*Lipogramma evides*, *Lipogramma haberi* and *Lipogramma levinsoni*), indicating perfect congruence between molecular and morphology-based species delimitation criteria. *Lipogramma trilineata* and *Lipogramma klayi*, which have two of the shallowest depth ranges among *Lipogramma* species (Fig. 10), were recovered as sister species. This pair is sister to a larger clade comprising *Lipogramma anabantoides* + *Lipogramma levinsoni* (as sister species) and *Lipogramma evides* + *Lipogramma haberi* + two putative new species superficially resembling *Lipogramma robinsi*. Not surprising given their morphological similarity, *Lipogramma evides*, and *Lipogramma haberi* resolve as sister species. There is strong support for a clade comprising the four deepest-known species in our phylogeny—*Lipogramma evides*, *Lipogramma haberi*, and the two “*Lipogramma robinsi*” species (Figs 9, 10). Baldwin and Robertson (2014) found a similar evolutionary grouping of deep-water species in the serranid genus *Liopropoma*, and Tornabene et al. (2016a) found repeated invasions of deep-reef depths in the family Gobiidae with subsequent species radiations entirely within the deep-reef zone. *Lipogramma flavescens*, which also inhabits deep water (200–300 m, Fig. 10), may be part of this clade. A dark ocellus on the base of the soft dorsal fin appears to be a synapomorphy of the clade comprising *Lipogramma anabantoides*, *Lipogramma levinsoni*, *Lipogramma evides*, *Lipogramma haberi*, and the two “*Lipogramma robinsi*” species. Presence of this ocellus in *Lipogramma flavescens* and *Lipogramma regia* suggests that they may also belong to this group, but genetic samples of both are needed for phylogenetic analysis. *Lipogramma flavescens* may be closely related to *Lipogramma haberi*, as they share a narrow dark bar through the eye, yellow coloration, and low gill-raker count (15–16), and they inhabit similar deep-reef depths (152–233 m for *Lipogramma haberi*, 200–300 m for *Lipogramma flavescens*). If the evolutionary relationships of *Lipogramma* species are correlated with depth as our data suggest, and if *Lipogramma regia*, which is known only from depths < 100 m is a member of the clade diagnosed by a dark ocellus on the soft dorsal fin, it may be most closely related to *Lipogramma anabantoides* and *Lipogramma levinsoni*, which are known only from depths < 120 m (*Lipogramma anabantoides*) and < 154 m (*Lipogramma levinsoni*). Those three are the only known *Lipogramma* species with a modal pectoral-fin count of 17 (Gilmore and Jones 1988: Table 2, this study). We note that the addition to our molecular phylogeny of the four known species of *Lipogramma* that are currently unavailable for analysis (*Lipogramma flavescens*, *Lipogramma regia*, *Lipogramma rosea*, and *Lipogramma robinsi*) could change our hypotheses of relationships within the genus.

The two “*Lipogramma robinsi*” included here (Table 3, Figs 9, 10) are genetically distinct and superficially different from one another and from *Lipogramma robinsi* Gilmore, 1997. A more thorough investigation of those three taxa is in progress, after which a key to all *Lipogramma* species will be constructed.

*Lipogramma* is currently classified along with *Gramma* in the family Grammatidae based on a single synapomorphy in the arrangement of cheek musculature (Gill and Mooi 1993). Molecular data have failed to corroborate the monophyly of the Grammatidae (Betancur-R et al. 2013, Mirande 2016); rather, those data suggest that *Lipogramma* and *Gramma* are each related to different taxa within the diverse Ovalentaria. Relationships within the Ovalentaria have proven difficult to resolve with traditional molecular markers (Betancur-R et al. 2013), molecular markers plus some morphological characters (Mirande 2016), and phylogenomic data (Eytan et al. 2015). Potential close relatives of *Lipogramma* based on molecular data include blennioids, cichlids, plesiopids, pseudochromids, and *Pholidichthys*. Some of these groups have been previously linked to either *Lipogramma*, *Gramma*, or both, based on shared morphological characters, but the homologies of many of these characters have been questioned (Gill and Mooi 1993). At present, the phylogenetic position of *Lipogramma* is unresolved.

**Figure 11.**
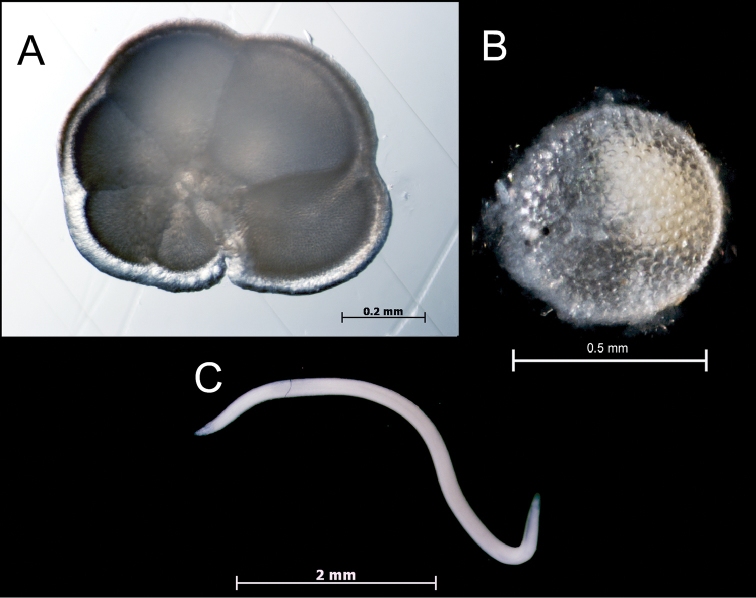
Items from stomachs of deep-reef *Lipogramma*: **A** Planktonic foraminiferan, possibly *Globorotalia manardii*, from *Lipogramma evides*, USNM 434771, collected at 174 m **B** Diatom, possibly *Coscinodiscus eccentricus*, from *Lipogramma levinsoni* sp. n., USNM 406140, collected between 137 and 146 m **C** Parasitic Nematoda from same specimen as **B**. Photos by A. Nonaka and L. Tornabene.

**Ecology and life history.** Little is known about community structure on deep reefs, including food networks. Although an analysis of the diet of banded basslets based on stomach contents is beyond the scope of this study, the gastrointestinal tract of the cleared and stained *Lipogramma evides* (USNM 434771) contained numerous individuals of a planktonic foraminiferan that was tentatively identified by Smithsonian Curator of Planktic Foraminifera Brian Huber as *Globorotalia manardii* (d’Orbigny) – Fig. 11A. Two items found in the gastrointestinal tract of *Lipogramma levinsoni* (USNM 406140) appear to be a diatom (possibly *Coscinodiscus eccentricus* Ehrenberg, Huber pers. comm, Fig. 11B) and a parasitic nematode (identification by Smithsonian Curator of Invertebrate Zoology Jon Norenberg and Assistant Professor of Biology at Virginia Military Institute Ashleigh Smythe, Fig. 11C). Future investigations of diets of deep-reef fish species are planned.

The broad Caribbean distributions of *Lipogramma levinsoni* and *Lipogramma evides* (Table 5) suggest a pelagic larval stage capable of dispersal, so it is perplexing that there are no records of *Lipogramma* or *Gramma* larvae from plankton tows (Asoh and Yoshikawa 1996, Hardy 2006). Thresher (1980) noted that *Lipogramma trilineata* constructs nests within algae in aquaria settings, and Asoh and Yoshikawa (1996) described similar nesting behavior in *Gramma loreto* Poey, 1868. The apparent restricted geographic distribution of *Lipogramma haberi* (Table 5) could indicate that some species have limited dispersal capabilities; however, the paucity of faunal investigations of deep-reef ecosystems may mask a larger geographic distribution for that species. Interestingly, Leis et al. (2012) calculated swimming speed for reared *Gramma loreto* larvae and found that the actual and relative critical speed (Ucrit) were so low that for most of the pelagic larval duration their ability to influence their dispersal by horizontal swimming would be much less than that of many other tropical fish species. Further study of the early life history of *Lipogramma* is needed, including exploring the possibility that planktonic dispersal in the genus may be limited.

## Conclusions

Adults and juveniles of the banded basslet, *Lipogramma evides*, were previously recognized as different ontogenetic color patterns of a single species. This study shows that the juvenile color pattern belongs to a cryptic species, described here as *Lipogramma levinsoni*. This study also documents the first known juveniles of *Lipogramma evides*, which share the color pattern of adults. A second new species that is morphologically similar to *Lipogramma evides*, *Lipogramma haberi*, is also described. These three basslet species are confined to deep-reef depths, but they stratify such that *Lipogramma levinsoni* occurs at shallower depths than *Lipogramma evides* and *Lipogramma haberi*. A molecular analysis of evolutionary relationships among available *Lipogramma* species reveals correlations between depth of occurrence and phylogeny, an eco-evolutionary pattern observed in other deep-reef Caribbean fishes that warrants further investigation. The two new basslets represent the eleventh and twelfth new fish species described in recent years from exploratory submersible diving by the Smithsonian’s Deep Reef Observation Project (DROP) to Caribbean depths of 300 m (Baldwin and Robertson 2013, 2014, 2015; Baldwin and Johnson 2014; Baldwin et al. 2016; Tornabene et al. 2016b, 2016c). Numerous other new fish and invertebrate species discovered by DROP await description, including the two putative new species identified in this study as morphologically similar to but distinct from *Lipogramma robinsi*. Considerably more effort is needed to adequately explore tropical deep reefs, diverse but largely overlooked global ecosystems.

## Supplementary Material

XML Treatment for Lipogramma levinsoni

XML Treatment for Lipogramma haberi

## Appendix 1.

**Table:** Links between DNA voucher specimens, GenBank accession numbers, and DNA sequences of Lipogramma derived for use in this study.

Catalog number	Tissue number	Species	GenBank COI	GenBank H3	GenBank TMO-4C4	GenBank Rag1	GenBank Rhodopsin	GenSeq designation
Photo Voucher Only	BAH10150	*Lipogramma anabantoides*	KX713732	KX713823	KX713880	KX713842	KX713862	genseq-5
USNM 413759	BAH9160	*Lipogramma anabantoides*		KX713824	KX713881	KX713843	KX713863	genseq-4
USNM 420334	BLZ5340	*Lipogramma anabantoides*	KX713733	–	–	–	–	genseq-4
USNM 414886	CUR12013	*Lipogramma evides*	KX713750	–	–	–	–	genseq-4
USNM 414889	CUR12031	*Lipogramma evides*	KX713751	KX713834	KX713891	KX713852	KX713872	genseq-4
USNM 414883	CUR12044	*Lipogramma evides*	KX713752	–	–	–	–	genseq-4
USNM 414884	CUR12050	*Lipogramma evides*	KX713753	–	–	–	–	genseq-4
USNM 414887	CUR12078	*Lipogramma evides*	KX713754	–	–	–	–	genseq-4
USNM 414890	CUR12084	*Lipogramma evides*	KX713755	–	–	–	–	genseq-4
USNM 414888	CUR12116	*Lipogramma evides*	KX713757	KX713835	KX713892	KX713853	KX713873	genseq-4
USNM 414882	CUR12118	*Lipogramma evides*	KX713758	–	–	–	–	genseq-4
USNM 414878	CUR12276	*Lipogramma evides*	KX713760	–	–	–	–	genseq-4
USNM 414881	CUR12280	*Lipogramma evides*	KX713761	–	–	–	–	genseq-4
USNM 414885	CUR12281	*Lipogramma evides*	KX713762	KX713837	KX713894	KX713855	KX713875	genseq-4
USNM 414879	CUR12288	*Lipogramma evides*	KX713763	–	–	–	–	genseq-4
USNM 414876	CUR12353	*Lipogramma evides*	KX713767	–	–	–	–	genseq-4
USNM 421602	CUR13100	*Lipogramma evides*	KX713771	–	–	–	–	genseq-4
USNM 426769	CUR13233	*Lipogramma evides*	KX713779	–	–	–	–	genseq-4
USNM 426770	CUR13234	*Lipogramma evides*	KX713780	–	–	–	–	genseq-4
USNM 426771	CUR13265	*Lipogramma evides*	KX713781	–	–	–	–	genseq-4
USNM 426737	CUR13266	*Lipogramma evides*	KX713782	–	–	–	–	genseq-4
USNM 426746	CUR13279	*Lipogramma evides*	KX713785	–	–	–	–	genseq-4
USNM 426709	CUR13286	*Lipogramma evides*	KX713786	–	–	–	–	genseq-4
USNM 426722	CUR13294	*Lipogramma evides*	KX713787	–	–	–	–	genseq-4
Photo Voucher Only	CUR15032	*Lipogramma evides*	KX713793	–	–	–	–	genseq-5
Photo Voucher Only	CUR15055	*Lipogramma evides*	KX713795	–	–	–	–	genseq-5
Photo Voucher Only	CUR15057	*Lipogramma evides*	KX713796	–	–	–	–	genseq-5
Photo Voucher Only	CUR15060	*Lipogramma evides*	KX713798	–	–	–	–	genseq-5
Photo Voucher Only	CUR15061	*Lipogramma evides*	KX713799	–	–	–	–	genseq-5
USNM 434771	CUR15091	*Lipogramma evides*	KX713811	–	–	–	–	genseq-4
USNM 434783	CUR15103	*Lipogramma evides*	KX713813	–	–	–	–	genseq-4
USNM 434784	CUR15104	*Lipogramma evides*	KX713814	–	–	–	–	genseq-4
USNM 431313	TIK003	*Lipogramma evides*	KX713822	–	–	–	–	genseq-4
USNM 422670, paratype	CUR13158	*Lipogramma haberi*	KX713775	–	–	KX713860	–	genseq-2
USNM 422679, holotype	CUR13171	*Lipogramma haberi*	KX713776	–	–	KX713861	–	genseq-1
USNM 434772, paratype	CUR15092	*Lipogramma haberi*	KX713812	–	–	–	–	genseq-2
USNM 406013	CUR11013	*Lipogramma klayi*	KX713737	KX713826	KX713883	KX713845	KX713865	genseq-3
USNM 406133	CUR11133	*Lipogramma klayi*	KX713740	KX713828	KX713885	KX713847	KX713867	genseq-3
USNM 406134	CUR11134	*Lipogramma klayi*	KX713741	–	–	–	–	genseq-3
USNM 406375	CUR11375	*Lipogramma klayi*	KX713744	–	–	–	–	genseq-3
USNM 406376	CUR11376	*Lipogramma klayi*	KX713745	KX713830	KX713887	KX713849	KX713869	genseq-3
USNM 422669	CUR13112	*Lipogramma klayi*	KX713772	–	–	–	–	genseq-3
USNM 422676	CUR13113	*Lipogramma klayi*	KX713773	–	–	–	–	genseq-3
USNM 422690	CUR13114	*Lipogramma klayi*	KX713774	–	–	–	–	genseq-3
Photo Voucher Only	CUR15064	*Lipogramma klayi*	KX713800	–	–	–	–	genseq-5
Photo Voucher Only	CUR15066	*Lipogramma klayi*	KX713801	–	–	–	–	genseq-5
Photo Voucher Only	CUR15068	*Lipogramma klayi*	KX713802	–	–	–	–	genseq-5
Photo Voucher Only	CUR15069	*Lipogramma klayi*	KX713803	–	–	–	–	genseq-5
Photo Voucher Only	CUR15070	*Lipogramma klayi*	KX713804	–	–	–	–	genseq-5
Photo Voucher Only	CUR15075	*Lipogramma klayi*	KX713805	–	–	–	–	genseq-5
Photo Voucher Only	CUR15076	*Lipogramma klayi*	KX713806	–	–	–	–	genseq-5
Photo Voucher Only	CUR15077	*Lipogramma klayi*	KX713807	–	–	–	–	genseq-5
Photo Voucher Only	CUR15084	*Lipogramma klayi*	KX713810	–	–	–	–	genseq-5
USNM 438687	DOM16036	*Lipogramma klayi*	KX713817	–	–	–	–	genseq-4
USNM 438741	DOM16090	*Lipogramma klayi*	KX713819	–	–	–	–	genseq-4
USNM 438742	DOM16091	*Lipogramma klayi*	KX713820	–	–	–	–	genseq-4
USNM 438803	DOM16152	*Lipogramma klayi*	KX713821	–	–	–	–	genseq-4
USNM 406011	CUR11011	*Lipogramma levinsoni*	KX713735	–	–	–	–	genseq-3
USNM 406012	CUR11012	*Lipogramma levinsoni*	KX713736	–	–	–	–	genseq-3
USNM 406018, paratype	CUR11018	*Lipogramma levinsoni*	KX713738	KX713827	KX713884	KX713846	KX713866	genseq-2
USNM 406019	CUR11019	*Lipogramma levinsoni*	KX713739	–	–	–	–	genseq-3
USNM 406139, holotype	CUR11139	*Lipogramma levinsoni*	KX713742	KX713829	KX713886	KX713848	KX713868	genseq-1
USNM 406140, paratype	CUR11140	*Lipogramma levinsoni*	KX713743	–	–	–	–	genseq-2
USNM 406393, paratype	CUR11393	*Lipogramma levinsoni*	KX713747	KX713832	KX713889	KX713851	KX713871	genseq-2
USNM 406394	CUR11394	*Lipogramma levinsoni*	KX713748	–	–	–	–	genseq-3
USNM 426784, paratype	CUR13183	*Lipogramma levinsoni*	KX713777	–	–	–	–	genseq-2
USNM 426754	CUR13184	*Lipogramma levinsoni*	KX713778	–	–	–	–	genseq-3
USNM 426774	CUR13267	*Lipogramma levinsoni*	KX713783	–	–	–	–	genseq-3
USNM 426730	CUR13268	*Lipogramma levinsoni*	KX713784	–	–	–	–	genseq-3
Photo Voucher Only	CUR15031	*Lipogramma levinsoni*	KX713792	–	–	–	–	genseq-5
Photo Voucher Only	CUR15058	*Lipogramma levinsoni*	KX713797	–	–	–	–	genseq-5
USNM 438703	DOM16052	*Lipogramma levinsoni*	KX713818	–	–	–	–	genseq-4
USNM 406392	CUR11392	*Lipogramma “robinsi*”	KX713746	KX713831	KX713888	KX713850	KX713870	genseq-4
USNM 406426	CUR11426	*Lipogramma “robinsi*”	KX713749	KX713833	KX713890	–	–	genseq-4
USNM 414913	CUR12101	*Lipogramma “robinsi*”	KX713756	–	–	–	–	genseq-4
USNM 414914	CUR12149	*Lipogramma “robinsi*”	KX713759	KX713836	KX713893	KX713854	KX713874	genseq-4
USNM 413864	CUR12290	*Lipogramma “robinsi*”	KX713764	KX713838	KX713895	KX713856	KX713876	genseq-4
USNM 414911	CUR12316	*Lipogramma “robinsi*”	KX713765	KX713839	KX713896	KX713857	KX713877	genseq-4
USNM 414912	CUR12317	*Lipogramma “robinsi*”	KX713766	KX713840	KX713897	KX713858	KX713878	genseq-4
USNM 430035	CUR13329	*Lipogramma “robinsi*”	KX713788	–	–	–	–	genseq-4
USNM 431687	CUR14079	*Lipogramma “robinsi*”	KX713789	–	–	–	–	genseq-4
USNM 431722	CUR14114	*Lipogramma “robinsi*”	KX713790	–	–	–	–	genseq-4
USNM 435299	CUR15012	*Lipogramma “robinsi*”	KX713791	–	–	–	–	genseq-4
USNM 436460	CUR15125	*Lipogramma “robinsi*”	KX713815	–	–	–	–	genseq-4
USNM 436474	CUR15139	*Lipogramma “robinsi*”	KX713816	–	–	–	–	genseq-4
Photo Voucher Only	BLZ8127	*Lipogramma trilineata*	JQ841643	–	–	–	–	genseq-5
Photo Voucher Only	BLZ8128	*Lipogramma trilineata*	JQ841642	–	–	–	–	genseq-5
USNM 415245	BLZ8168	*Lipogramma trilineata*	JQ841645	–	–	–	–	genseq-4
USNM 415298	BLZ8274	*Lipogramma trilineata*	JQ841646	KX713825	KX713882	KX713844	KX713864	genseq-4
Photo Voucher Only	BLZ8343	*Lipogramma trilineata*	JQ841644	–	–	–	–	genseq-5
USNM 404204	BLZWF204	*Lipogramma trilineata*	KX713734	–	–	–	–	genseq-4
USNM 414989	CUR13082	*Lipogramma trilineata*	KX713768	–	–	–	–	genseq-3
USNM 414990	CUR13089	*Lipogramma trilineata*	KX713769	–	–	–	–	genseq-3
USNM 414991	CUR13090	*Lipogramma trilineata*	KX713770	KX713841	KX713898	KX713859	KX713879	genseq-3
Photo Voucher Only	CUR15034	*Lipogramma trilineata*	KX713794	–	–	–	–	genseq-5
Photo Voucher Only	CUR15078	*Lipogramma trilineata*	KX713808	–	–	–	–	genseq-5
Photo Voucher Only	CUR15079	*Lipogramma trilineata*	KX713809	–	–	–	–	genseq-5

## Appendix 2.

**Specimens of *Lipogramma evides* examined in this study.**

ANSP 134329, holotype, 34.4 mm SL, R/V Pillsbury Sta. 581, Mexico, Arrowsmith Bank, 21°05'N, 86°23'W, 146–265 m depth, 22 May 1967; ANSP 134330, n=2, paratypes, 28.0–32.0 mm SL, R/V Pillsbury Sta. 581, Mexico, Arrowsmith Bank, 21 05'N, 86 23'W, 146–265 m depth, 22 May 1967; ANSP 134331, paratype, 17.2 mm SL, R/V Pillsbury Sta. 969, southeast of Barbuda, 17°27.8'N, 61°41.1'W, 68–216 m depth, 20 July 1969; FMNH 82583, paratype, 34.5 mm SL, Nicaragua, 12°32'N, 82°25 ‘ W, 155 m depth, 23 May 1692; USNM 426801 25.1 mm SL, Curasub submersible, sta. CURASUB13-18, Curaçao, Playa Forti, Westpoint, 12.3679 N, 69.1553 W, no depth data, 15 August 2013, C. Baldwin, B. Brandt, A. Schrier, K. Johnson & C. DeForest; USNM 431410, 12.7 mm SL , Curasub submersible, sta. CURASUB14-07, Curaçao, in between Porto Marie and Daaibooi beaches, 12.202842 N, 69.089507 W, 123 m depth, 21 March 2014, C. Baldwin et al.; USNM 426746, 45.4 mm SL, tissue no. CUR13279, Curasub submersible, sta. CURASUB13-19, Curaçao, Playa Forti, Westpoint, 12.3679 N, 69.1553 W, 179 m depth, 15 August 2013, B. Van Bebber, N. Knowlton, A. Schrier & R. Sant; USNM 431408, 35.5 mm SL, Curasub submersible, sta. CURASUB14-02, Curaçao, off Substation Curaçao downline., 12.083197 N, 68.899058 W, no depth data available, 17 March 2014, B. Brooks et al.; USNM 410992, 43.0 mm SL, Curasub submersible, sta. CURASUB13-33, Caracas Baii and back to Substation Curaçao downline, 12.068 N, 68.873367 W, 215 m depth, 5 November 2013, C. Baldwin, B. Brandt, A. Schrier & C. Castillo; UF 238591, 34.5 mm SL, Curasub submersible, sta. CURASUB15-13, Northwest corner of Klein Curaçao, 11.998453 N, 68.651308 W, 182 m depth, 28 August 2015, C. Baldwin & B. Van Bebber; USNM 434771, 33.3 mm SL, cleared and stained, tissue no. CUR15091, Curasub submersible, sta. CURASUB15-12, northwest corner of Klein Curaçao, 11.998453 N, 68.651308 W, 174 m depth, 27 August 2015, B. Brandt & A. Schrier; USNM 434783, 17.9 mm SL, tissue no. CUR15103, Curasub submersible, sta. CURASUB15-13, Northwest corner of Klein Curaçao, 11.998453 N, 68.651308 W, 171 m depth, 28 August 2015, C. Baldwin & B. Van Bebber; UF 238590, 27.7 mm SL, tissue no. CUR15104, Curasub submersible, sta. CURASUB15-13, Northwest corner of Klein Curaçao, 11.998453 N, 68.651308 W, 172 m depth, 28 August 2015, C. Baldwin & B. Van Bebber; USNM 431313, 39.2 mm SL, tissue no. T1K003, Curasub submersible, sta. CURASUB14-03, Curaçao, west of Substation Curaçao downline, 12.083197 N, 68.899058 W, 177 m depth, 18 March 2014, C. Baldwin et al.; USNM 414886, 24.9 mm SL, tissue no. CUR12013, Curasub submersible, sta. CURASUB12-01, Curaçao, off of Substation Curaçao downline, 12.083197 N, 68.899058 W, 171 m depth, 21 May 2012, C. Baldwin, A. Schrier & B. Brandt; USNM 414889, 31.3 mm SL, tissue no. CUR12031, Curasub submersible, Curaçao, off of Substation Curaçao downline, 12.083197 N, 68.899058 W, no depth data available, 21 May 2012, C. Baldwin et al.; USNM 414883, 40.0 mm SL, tissue CUR12044, Curasub submersible, Curaçao, off of Substation Curaçao downline, 12.083197 N, 68.899058 W, no depth data available, 21 May 2012, C. Baldwin et al.; USNM 414884, 32.0 mm SL, tissue no. CUR12050, Curasub submersible, sta. CURASUB12-11, Curaçao, off of Substation Curaçao downline, 12.083197 N, 68.899058 W, 164 m depth, 6 August 2012, B. Brandt, C. Baldwin, A. Schrier & A. Driskell; USNM 414887, 31.3 mm SL, tissue CUR12078, Curasub submersible, Curaçao, off of Substation Curaçao downline, 12.083197 N, 68.899058 W, no depth data available, 21 May 2012, C. Baldwin et al.; USNM 414890, 40.3 mm SL, tissue no. CUR12084, Curasub submersible, Curaçao, off of Substation Curaçao downline, 12.083197 N, 68.899058 W, no depth data available, 21 May 2012, C. Baldwin et al.; USNM 414888, 39.8 mm SL, tissue no. CUR12116, Curasub submersible, sta. CURASUB12-14, Curaçao, east of downline off Substation dock, 12.083197 N, 68.899058 W, 133 m depth, 9 August 2012, A. Schrier, B. Brandt, C. Castillo, A. Driskell & D. Robertson; USNM 414880, 29.0 mm SL, tissue no. CUR12117, Curasub submersible, sta. CURASUB12-14, Curaçao, east of downline off Substation dock, 12.083197 N, 68.899058 W, 134 m depth, 9 August 2012, A. Schrier, B. Brandt, C. Castillo, A. Driskell & D. Robertson; USNM 414882, 20.1 mm SL, tissue CUR12118, Curasub submersible, sta. CURASUB12-14, Curaçao, east of downline off Substation dock, 12.083197 N, 68.899058 W, 134 m depth, 9 August 2012, A. Schrier, B. Brandt, C. Castillo, A. Driskell & D. Robertson; USNM 414878, 24.1 mm SL, tissue no. CUR12276, Curasub submersible, sta. CURASUB12-16, Curaçao, west to Stella Maris and down, 154 m depth, 13 August 2012, A. Schrier, C. Baldwin & B. Van Bebber; USNM 414881, 21.1 mm SL, tissue no. CUR12280, 21.2 mm SL, Curasub submersible, sta. CURASUB12-17, Curaçao, East of downline off Substation Curaçao dock, 12.083197 N, 68.899058 W, 161 m depth, 13 August 2012, A. Schrier, B. Brandt, C. Castillo & D. Robertson; USNM 414885, 24.4 mm SL, tissue no. CUR12281, Curasub submersible, sta. CURASUB12-17, Curaçao, East of downline off Substation Curaçao dock, 12.083197 N, 68.899058 W, 161 m depth, 13 August 2012, A. Schrier, B. Brandt, C. Castillo & D. Robertson; USNM 414879, 25.0 mm SL, tissue no. CUR12288, Curasub submersible, sta. CURASUB12-16, Curaçao, west to Stella Maris and down, 154 m depth, 13 August 2012, A. Schrier, C. Baldwin & B. Van Bebber; USNM 414876, 41.7 mm SL, tissue no. CUR12353, Curasub submersible, no station data, off Substation Curaçao dock, 12.083197 N, 68.899058 W, no depth data available, 2012, Substation Curaçao crew; USNM 421602, 43.6 mm SL, tissue CUR13100, Curasub submersible, Curaçao, off Substation Curaçao, 12.083197 N, 68.899058 W, no depth data available; USNM 426769, 19.0 mm SL, tissue CUR13233, Curasub submersible, sta. CURASUB13-12, Curaçao, off downline at Substation Curaçao, 12.083197 N, 68.899058 W, 137–173 m depth, 7 August 2013, C. Baldwin, D. Robertson, C. Castillo & B. Van Bebber; USNM 426770, 13.7 mm SL, tissue no. CUR13234, Curasub submersible, sta. CURASUB13-12, Curaçao, off downline at Substation Curaçao, 12.083197 N, 68.899058 W, 137–173 m depth, 7 August 2013, C. Baldwin, D. Robertson, C. Castillo & B. Van Bebber; USNM 426771, 24.9 mm SL, tissue CUR13265, Curasub submersible, sta. CURASUB13-18, Curaçao, Playa Forti, westpoint, 12.3679 N, 69.1553 W, 137–164 m depth, 15 August 2013, C. Baldwin, B. Brandt, A. Schrier, K. Johnson & C. DeForest; USNM 426737, 19.3 mm SL, tissue CUR13266, Curasub submersible, sta. CURASUB13-18, Curaçao, Playa Forti, westpoint, 12.3679 N, 69.1553 W, 137–173 m depth, 15 August 2013, C. Baldwin, B. Brandt, A. Schrier, K. Johnson & C. DeForest; USNM 426709, 40.2 mm SL, tissue no. CUR13286, Curasub submersible, sta. CURASUB13-21, Curaçao, off Substation Curaçao downline, 12.083197 N, 68.899058 W, 171–201 m depth, 17 August 2013, C. Baldwin, A. Schrier, B. Brandt & A. Driskell; USNM 426722, 32.5 mm SL, tissue no. CUR13294, Curasub submersible, sta. CURASUB13-21, Curaçao, off Substation Curaçao downline, 12.083197 N, 68.899058 W, 171–201 m depth, 17 August 2013, C. Baldwin, A. Schrier, B. Brandt & A. Driskell.

## Supplementary materials

Supplementary material 1 Figure S1 Data type: Tif image file Explanation note: Neighbor-joining network based on COI sequences of *Lipogramma* species investigated in this study. Scale-bar units are substitutions per site. This dataset is made available under the Open Database License (http://opendatacommons.org/licenses/odbl/1.0/). The Open Database License (ODbL) is a license agreement intended to allow users to freely share, modify, and use this Dataset while maintaining this same freedom for others, provided that the original source and author(s) are credited.Carole C. Baldwin, D. Ross Robertson, Ai Nonaka, Luke Tornabene
