# Supplementary figures and images for: Two new deep-reef basslets (Teleostei, Grammatidae, Lipogramma), with comments on the eco-evolutionary relationships of the genus
Source: Zookeys. 2016 Dec 7;(638):45–82. doi: 10.3897/zookeys.638.10455 (PMC5270743; doi:10.3897/zookeys.638.10455)

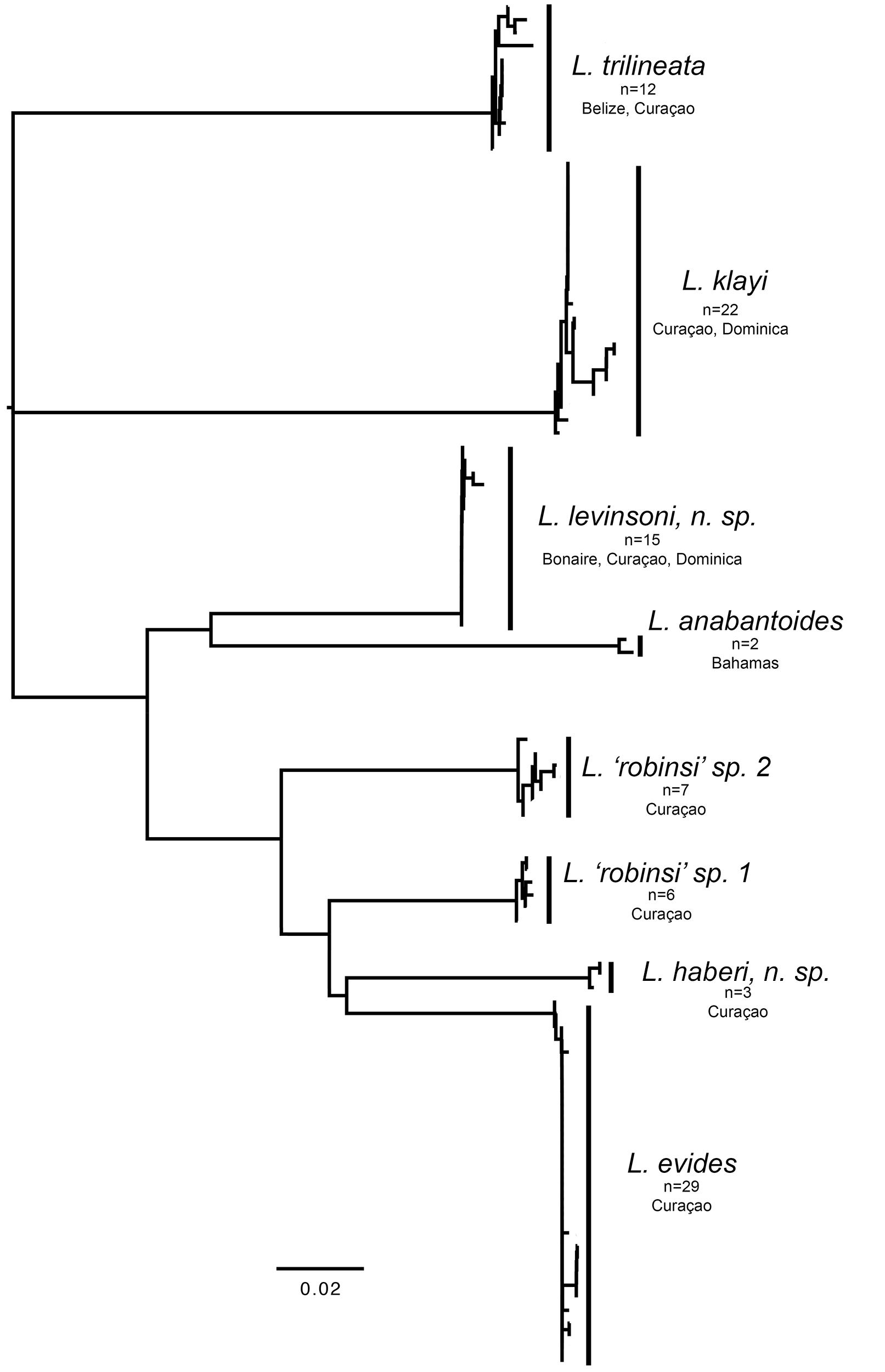

Supplement: Supplementary material 1 — Figure S1 [file zookeys-638-045-s001.tif]
